# Mechanisms by Which Skeletal Muscle Myokines Ameliorate Insulin Resistance

**DOI:** 10.3390/ijms23094636

**Published:** 2022-04-22

**Authors:** Rekha Balakrishnan, Debbie C. Thurmond

**Affiliations:** Department of Molecular and Cellular Endocrinology, Arthur Riggs Diabetes and Metabolism Research Institute, City of Hope Beckman Research Institute, 1500 E. Duarte Road, Duarte, CA 91010, USA; rbalakrishnan@coh.org

**Keywords:** myokines, insulin resistance, type 2 diabetes, metabolic disorder, inter-organ cross-talk

## Abstract

The skeletal muscle is the largest organ in the body and secretes circulating factors, including myokines, which are involved in various cellular signaling processes. Skeletal muscle is vital for metabolism and physiology and plays a crucial role in insulin-mediated glucose disposal. Myokines have autocrine, paracrine, and endocrine functions, serving as critical regulators of myogenic differentiation, fiber-type switching, and maintaining muscle mass. Myokines have profound effects on energy metabolism and inflammation, contributing to the pathophysiology of type 2 diabetes (T2D) and other metabolic diseases. Myokines have been shown to increase insulin sensitivity, thereby improving glucose disposal and regulating glucose and lipid metabolism. Many myokines have now been identified, and research on myokine signaling mechanisms and functions is rapidly emerging. This review summarizes the current state of the field regarding the role of myokines in tissue cross-talk, including their molecular mechanisms, and their potential as therapeutic targets for T2D.

## 1. Introduction

### 1.1. Diabetes and Skeletal Muscle Insulin Resistance

Diabetes mellitus has a complex pathophysiology that combines impaired metabolism and deficient glucose disposal; it affects multiple organs and increases the risk of life-threatening cardiomyopathy, as well as complications of nephropathy, neuropathy, and retinopathy [1,2,3,4]. The skeletal muscle is the largest organ in the body and is essential to maintain vital functions such as movement, postural support, breathing, and thermogenesis [5]. Notably, skeletal muscle is also a primary site for glucose uptake; indeed, euglycemic hyperinsulinemic clamp experiments demonstrate that 80–90% of infused glucose is taken up by skeletal muscle [6]. Diabetes mellitus is broadly divided into type 1 (T1D) and type 2 (T2D) diabetes. T1D is a chronic autoimmune disorder in which dysfunctional pancreatic islet β-cells are targeted for destruction, thereby depleting insulin and impairing glucose uptake by peripheral tissues such as skeletal muscle and fat. This dysfunction results in persistent high circulatory glucose levels. In T2D, which accounts for about 90% of all diabetes cases, peripheral organs, including skeletal muscle, fat, and the liver, become insulin resistant, thereby leading to poor glucose clearance and high circulatory glucose levels. As skeletal muscle is the predominant site of postprandial glucose clearance, skeletal muscle insulin resistance is thought to be the major underlying cause of T2D. The persistently higher levels of circulating glucose in T2D signal pancreatic islet β-cells to produce more insulin, and eventually, the overworked β-cells become dysfunctional and insulin secretion is impaired. Thus, the skeletal muscle and pancreatic β-cells are central regulators of glucose homeostasis in the body.

Insulin resistance, also known as prediabetes, is an intermediate metabolic state between normoglycemia and T2D, wherein impaired fasting glucose and/or impaired glucose tolerance leads to metabolic dyshomeostasis. Within approximately five years of diagnosis, prediabetic individuals have an about 50% chance of developing T2D and other metabolic complications that ultimately decrease their lifespan [7].

### 1.2. Current Therapies for Prediabetes and Disease Management

There are currently 536.6 million people worldwide with diabetes, and this is expected to increase to an estimated 738.2 million by 2045 [8]. In the US, more than 37.3 million people of all ages (~11.3% of the population) have diabetes and 1.5 million people are newly diagnosed with T2D every year [9]. Furthermore, adults with prediabetes constitute 34.5% of the US population, indicating that hyperglycemia and its associated metabolic anomalies will continue to be a serious health concern. Current treatments mostly focus on lifestyle and drug-based interventions [10,11,12]. However, long-term lifestyle modifications often fail, and the current anti-diabetes drugs can trigger serious adverse events including heart failure, hepatotoxicity, and obesity [13,14]. Due to these limitations, there is a considerable effort underway to identify and develop novel therapeutics to reverse and mitigate prediabetes. This requires a multi-pronged approach since the pathophysiology involves β-cell dysfunction, skeletal muscle insulin resistance, and contributions from other peripheral organs. Thus, research focusing on understanding skeletal muscle insulin sensitivity, energy metabolism, and the role of myokines with endocrine functions will identify potent candidate therapies in future.

### 1.3. Skeletal Muscle Myokine-Mediated Regulatory Actions

Skeletal muscle secretes numerous myokines, which are defined as cytokines and peptides that are produced and released by muscle fibers. Myokines are involved in the autocrine regulation of metabolism in muscles and the para/endocrine regulation of other organs that express myokine receptors, including the pancreas, adipose tissue, liver, heart, bone, gut, and brain [15,16,17]. For instance, myokines produced by muscles during contraction can improve insulin sensitivity and glucose oxidation via autocrine action [18]. Furthermore, muscle fiber-derived myokines are involved in the autocrine/paracrine regulation of satellite cells and promote muscle hypertrophy during exercise [19,20]. Myokines involved in metabolic regulation can also ameliorate multiple diseases including insulin resistance, obesity, and cancer [21,22,23,24,25]. Over 3000 possible myokines have been identified in humans and rodents [26]. Interestingly, the functions of more than 100 myokines, including many novel ones, from the secretomes of primary human myotubes [27,28,29] and murine myocytes [28,30,31] have been determined. 

Dysfunctional myokine secretion plays a role in the pathogenesis of aging and metabolic diseases, including obesity, T2D, and sarcopenia [32,33,34]. Aging is associated with decreases in the secretion of beneficial myokines in rodents and humans, such as Apelin, Decorin, β-Aminoisobutyric acid (BAIBA), Sesterin, Secreted protein acidic and rich in cysteine (SPARC), Interleukin-15 (IL-15), and Irisin [35,36,37,38,39,40]. Furthermore, increased levels of the detrimental myokine, myostatin, is found at higher levels in streptozotocin-induced T1D mice and in the serum of patients with T1D and T2D [41,42,43]. Moreover, myostatin inhibition by adeno-associated virus-induced overexpression of the myostatin propeptide in mice increased the skeletal muscle glucose uptake in insulin-resistant HFD-fed mice. Myostatin also suppresses muscle regeneration, and this pathological effect is partially reversed by regular exercise and physical activity [44]. 

Myokines may be critical regulators of age-related pathologies including diabetes, muscular atrophy, and chronic inflammation. Indeed, serum from T2D patients contains reduced levels of beneficial myokines such as Irisin, IL-13, and FSTL-1 [45]. Interestingly, myokines secreted by myotubes impact β-cell function, proliferation, and survival; myokines from healthy myotubes act in a beneficial way, while myokines from insulin-resistant myotubes act in a detrimental way, suggesting that skeletal muscle-to-pancreas cross-talk regulates insulin secretion [46]. Similarly, the myokine expression pattern in the secretome of T2D patients differs from that of healthy individuals, and proteomic analysis from human primary skeletal muscle cells isolated from T2D patients shows altered myokine profiles compared to skeletal muscle cells from healthy donors [47]. Therefore, the focus of this review is to highlight recent advances in skeletal muscle inter-organ cross-talk mechanisms that impact whole-body glucose homeostasis, incorporating thoughts on how this new knowledge might be leveraged to ameliorate insulin resistance for the prevention and treatment of T2D.

## 2. Myokine-Mediated Muscle-to-Muscle and Muscle-to-Pancreas Communication

The evidence described in the prior section indicates that skeletal muscle can communicate with other organs through myokines secreted into the bloodstream during muscle contraction. Moreover, some of the beneficial circulating myokines involved in metabolic regulation are downregulated in T2D individuals. Hence, it is no surprise that common risk factors such as a sedentary lifestyle and obesity are correlated with decreased muscle contraction, impaired energy metabolism, and insulin resistance. Therefore, strategies to improve/regulate myokine release and function could present therapeutic opportunities to prevent and/or reverse T2D.

### Myokines Mediate Muscle-to-Muscle Cross Talk

**Figure 1:**
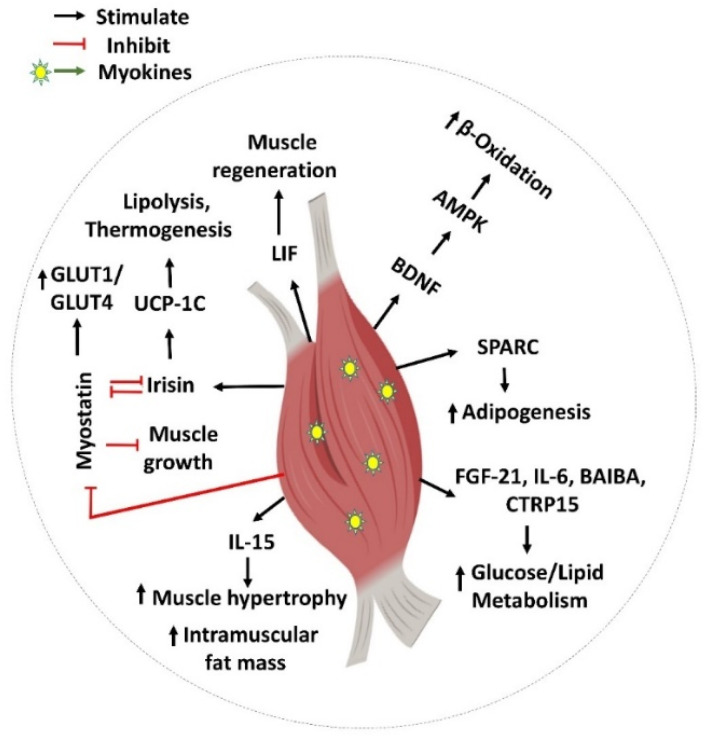
Myokine-mediated regulation of skeletal muscle function. Myokines such as SPARC, FGF-21, IL-6, BAIBA, CTRP15, BDNF, LIF, Irisin, myostatin (GDF-8), and IL-15 are involved in various biological processes including muscle generation, adipogenesis, muscle hypertrophy, muscle growth, and glucose and lipid regulation locally inside the skeletal muscle. This figure was created with Biorender.com.

Exercise is a proven lifestyle intervention for the treatment of T2D. Improved insulin sensitivity and glucose disposal is the well-known underlying molecular mechanism for the benefits of physical activity on T2D. Myokines released during or after exercise, which can exert effects locally within the muscle, are emerging as key mechanisms for these muscle metabolic modifications (Figure 1). Most of these secreted myokines influence metabolism, and/or are involved in muscle regeneration, satellite cell proliferation, and hypertrophic responses. Therefore, myokines are important for regulating skeletal muscle homeostasis and its adaptation to exercise training.

**FGF21:** Fibroblast growth factor 21 (FGF21) is a myokine with multiple therapeutic benefits against obesity-related medical complications [48]. The activity of FGFs is mediated by their binding to FGF receptors (FGFRs) and the co-receptor β-Klotho (KLB) [49,50]. In vivo gene knockout and activating antibodies for FGFR1 or the FGFR1/KLB complex determined that the FGFR1C isoform is an important target of FGF21′s function [51,52,53]. FGF21 expression in human skeletal muscle is reported to be activated during hyperinsulinemia, and thus it has been classified as a novel insulin-stimulated myokine [54]. FGF21 mRNA and protein levels were reported to be increased in the gastrocnemius muscle and serum of skeletal muscle-specific AKT1-overexpressing mice. In addition, AKT-enriched C2C12 myotubes showed elevated FGF21 expression [55]. Both of these results indicate that FGF21 secretion by skeletal muscle is regulated by the phosphatidylinositol 3-kinase (PI3-kinase)/AKT1 signaling pathway.

FGF21 regulates glucose and lipid metabolism and helps in maintaining energy balance. In support of this notion, FGF21 injection lowers fasting glucose, triglycerides, insulin, and glucagon levels in obese diabetic rodents [56,57] and rhesus monkeys [58,59]. Furthermore, chronic administration of FGF21 analogs ameliorates dyslipidemia and reduces body weight in obese and T2D patients, and also decreases fasting insulin levels while increasing adiponectin levels [60,61]. Acting via AMPK regulation, FGF21 protects against atrophy-induced inflammation, and its deficiency induces inflammation and worsens the obesity-induced atrophy of skeletal muscle [62]. Thus, overall, FGF21 is an insulin-stimulated beneficial myokine that regulates energy metabolism and protects against chronic metabolic disorders such as T2D and obesity.

**Irisin:** Irisin is a beneficial myokine secreted by contracting skeletal muscle into the circulation after proteolytic cleavage from its precursor, fibronectin type III domain-containing protein 5 (FNDC5) [63]. Mice overexpressing FNDC5 exhibited protection from high fat diet (HFD) diet-induced insulin resistance [64]. FNDC5 is regulated by a peroxisome proliferator-activated receptor γ coactivator 1-α (PGC1α) [65], a master regulator of genes involved in metabolism, thermogenesis, and antioxidant defense. In response to exercise, PGC1α expression and activity levels are elevated, and it coordinates the regulation of nuclear- and mitochondrial-encoded genes needed for contractile and metabolic adaptations in skeletal muscle [66,67,68]. Consistent with this, FNDC5 protein expression was increased in muscle obtained from exercise-trained rodents and humans, whilst plasma Irisin levels were shown to be increased in mice and humans after endurance exercise [64]. In addition, using adenoviral overexpression of FNDC5, the same study had reported that Irisin increases total body energy expenditure and protects against obesity-induced insulin resistance in mice.

Moreover, recent clinical studies have shown that circulating Irisin levels are reduced in T2D patients [69,70]. Consistent with this, ex vivo Irisin treatment improved the insulin-stimulated glucose uptake in muscle cells exposed to a lipotoxic T2D-mimicking milieu containing high palmitate levels [71]. Irisin’s effects are mediated by AMPK activation, which triggers p38 MAPK signaling and GLUT4 vesicle trafficking to the plasma membrane [72,73]. Despite many reported beneficial effects, the receptor for Irisin still remains unknown in most of the tissues except osteocytes, adipocytes, and enterocytes where αVβ5 integrin is determined as the Irisin receptor [74]. Overall, it has been reported that Irisin regulates glucose metabolism in skeletal muscle in an autocrine manner [73]. Given that Irisin also has positive effects in physiological functions such as thermogenesis, and glucose- and lipid-oxidation, it carries potential to be an attractive target for treating metabolic disorders.

**SPARC:** Secreted protein acidic and rich in cysteine (SPARC)/osteonectin is an exercise-responsive myokine. It has been reported that exercise-induced changes in muscle performance (metabolic strength and development), including lactate-induced changes, are SPARC-dependent [75]. For example, whole-body SPARC knockout mice exhibited an impaired metabolism and defective phosphorylation of AMPK and protein kinase B in the skeletal muscle. Consistent with this, treatment with SPARC (injected intraperitoneally with recombinant SPARC protein) improved glucose tolerance and activated AMPK in the skeletal muscle of SPARC knockout mice [76]. In addition, SPARC treatment to HFD-induced obese mice reversed their glucose intolerance and restored skeletal muscle AMPK signaling. SPARC deficiency in mice also decreases skeletal muscle mass and increases age-dependent adiposity, as skeletal muscle mass changes are inversely correlated with adipose mass changes [77]. In cultured myoblasts, SPARC treatment induces myogenic differentiation [78,79]. SPARC gene expression is reduced during aging, which may be related to observed age-related decreases in the levels of skeletal muscle progenitor cells [80]. Overall, SPARC is a beneficial myokine that is involved in AMPK-mediated glucose regulation and improves glucose tolerance. 

**BAIBA:** Known also as 3-amino-2-methylpropanoic acid, BAIBA is a small molecule catabolite of thymine and valine metabolism in mammals, which is produced by and secreted from skeletal muscle. BAIBA is a novel protective myokine that is increased during exercise via a PGC1α-dependent mechanism, improves insulin sensitivity, and protects against HFD-induced obesity [81,82]. Similar to other myokines, BAIBA enrichment/overexpression increases fatty acid oxidation and decreases lipogenesis in mice, resulting in a reduced body fat percentage [83]. BAIBA is produced in skeletal muscle during exercise and protects against obesity-dependent metabolic disorders, including T2D and non-alcoholic fatty liver disease [84,85]. BAIBA treatment of palmitate-exposed C2C12 myocytes and the skeletal muscle of HFD-fed mice ameliorated defects in the insulin receptor substrate (IRS)-1/Akt-mediated insulin signaling pathway. In addition, BAIBA infusion reversed HFD-induced weight gain and improved glucose tolerance in mice. BAIBA also suppressed inhibitory κBα (IκBα) phosphorylation, nuclear factor κB (NFκB) nuclear translocation, whilst promoting AMPK phosphorylation and the expression of peroxisome proliferator-activated receptor gamma (PPARδ) in mouse skeletal muscle and C2C12 cells [82]. Thus, BAIBA treatment protects against insulin resistance, prevents inflammation, and improves β-oxidation in skeletal muscle via the AMPK-PPARδ pathway. As with most other myokines discussed so far, BAIBA also communicates in a paracrine fashion, whereby it enhances the browning of white adipose tissue and increases β-oxidation in the liver through mechanisms mediated by peroxisome proliferator-activated receptor α (PPARα) [83]. Thus, BAIBA treatment prevents HFD-induced obesity through improving glucose tolerance, β-oxidation, and suppressing inflammatory pathways [81,86]. 

**Brain-derived neurotrophic factor (BDNF):** Protein and mRNA levels of BDNF are increased in human skeletal muscle after exercise [87]. BDNF is abundantly expressed in slow twitch skeletal muscle fibers, and its beneficial effects in skeletal muscle are mediated through AMPKα-PGC1α-mediated mitochondrial function and β-oxidation [88]. BDNF initiates its beneficial effects by binding to the tropomyosin-related kinase receptor B (TrkB), which subsequently activates phosphoinositide-3-kinase (PI3K)/Akt, Ras/extracellular signal-regulated kinase (ERK), and phospholipase C (PLCγ)/protein kinase C (PKC) signaling pathways [89]. Skeletal muscle specific BDNF knockout mice have impaired glucose to fatty acid utilization during fasting, linked to reduced muscle strength, myofiber necrosis and insulin resistance [90]. Interestingly, skeletal muscle-specific BDNF regulates the glycolytic muscle fiber type and metabolism [91]. BDNF addition to C2C12 myotubes correlates with a high mitochondrial DNA content and increased β-oxidation rate, facilitating mitochondrial fatty acid transport. Similarly, chronic subcutaneous or intracerebroventricular administration of BDNF increased muscle glucose uptake and enhanced energy expenditure in obese diabetic C57BL/KsJ-db/db mice [92]. Together, these pieces of evidence indicate that BDNF signaling is vital for balancing glucose and lipid metabolism in skeletal muscle. 

**Interleukin-6 (IL-6):** IL-6 is synthesized by and released from skeletal muscle in large amounts during physical activity, classifying it as a myokine. However, disparate reports of IL-6 contributing to positive and negative actions have led to controversy. For example, one finding that IL-6 pre-treatment in mice improves skeletal muscle glucose uptake, as assessed by hyperinsulinemic-euglycemic clamp analysis [93], supports the concept that IL-6 plays a positive role in skeletal muscle. In addition, at 3 months of age, IL-6 knockout mice showed an impaired exercise capacity and glucose intolerance, and they became obese by 9 months; however, these anomalies were linked to decreased levels of AMPK, making it unclear whether IL-6 was the causative factor [94]. Consistent with a beneficial effect of IL-6, in humans, IL-6 injection stimulated GLUT4 translocation and improved skeletal muscle insulin sensitivity [95]. Counterintuitively, IL-6 levels can be found elevated in insulin resistance and T2D. In addition, palmitate-induced IL-6 production was associated with a decreased glucose uptake in myocytes; this was reversed by an anti–IL-6 antibody [96,97]. Further confounding the interpretation of IL-6 function, IL-6 production is stimulated by TNFα and was initially found to be elevated in T2D [98], yet a recent human study found no changes in the circulating levels of IL-6 in T2D patients compared to control subjects [99]. Overall, IL-6 is stimulated by physical activity, but its effect on T2D is less clear, with evidence of both positive and negative actions.

**Leukemia inhibitory factor (LIF):** LIF is produced by and released from skeletal muscle cells [100]. Recombinant human LIF induces myoblast proliferation, and LIF mRNA and protein levels were found to be upregulated in contracting cultured human myotubes isolated from muscle biopsies of the vastus lateralis muscle, as well as in human skeletal muscle after resistance exercise [101]. LIF activates the transcription factors Jun-B and c-Myc, which promote satellite cell proliferation in an autocrine or paracrine fashion [101]. LIF was also found to increase the phosphorylation of AKT at Ser^473^ in soleus and extensor digitorum longus muscles and increase glucose uptake in both oxidative and glycolytic muscles [102]. Counterintuitively, LIF protein and its receptor (LIFR) are also elevated in muscle tissue and cultured myoblasts from T2D individuals, but LIF-stimulated cell proliferation is impaired in diabetic myoblasts [103,104]. Given that others have reported that LIF is immediately secreted and does not accumulate in skeletal muscle [105], it remains possible that these disparate findings could be caused by secretion defects in diabetic individuals rather than increased LIF biosynthesis. Experiments that distinguish these possibilities will be important to gain a deeper understanding of the interplay between LIF and metabolic disease. 

**Interleukin-15 (IL-15):** Skeletal muscle is an important source of circulatory IL-15 levels. IL-15 is a member of the IL-2 superfamily and, in humans and mouse models, IL-15 levels increase after acute physical exercise [35,106,107,108]. IL-15 is associated with beneficial actions; for instance, IL-15 overexpression induces weight loss and reduces white adipose tissue mass in rodents [95,109,110]. Moreover, enrichment of IL-15 protects against HFD-induced obesity and insulin resistance in mice models [111,112]. Consistent with this, obese human subjects have decreased levels of circulating IL-15 compared to lean individuals [95]. However, although IL-15 treatment of C2C12 myotubes increases GLUT4 gene expression and GLUT4 vesicle translocation, glucose uptake is not coordinately increased [113,114]. Instead, the effect of IL-15 is likely to occur at the level of the muscle tissue. In rodents, increased levels of circulating IL-5 induced fiber-type shifts, which promote an oxidative phenotype with increased mitochondrial DNA levels and cytochrome C oxidase activity [35,115]. Indeed, IL-15 therapy was found to mimic the anti-aging effects of exercise on skeletal muscle and skin in mouse models, suggesting it is a beneficial strategy to attenuate aging [35]. Furthermore, IL-15 treatment of skeletal muscle cells was found to exert protection against H_2_O_2_-induced oxidative stress and enhance mitochondrial function through a PPARδ-dependent mechanism. Overall, IL-15 may act in an auto/paracrine manner that is responsible for the skeletal muscle-mediated positive effects of exercise. Collectively, this evidence suggests that increasing IL-15 expression is a candidate intervention to prevent and remediate obesity and T2D.

**Myonectin (CTRP15):** Myonectin is a recently discovered mycophenolate that is released by skeletal muscle. It belongs to the C1q/TNF-related protein (CTRP) family, which is involved in the regulation of glucose and fatty acid metabolism [116,117,118]. Amongst the CTRP family members, myonectin is the one whose expression is limited only to skeletal muscle [119]. Moreover, slow-twitch fibers with higher oxidative metabolism express higher levels of the myonectin gene relative to fast-twitch fibers, which have a higher glycolytic metabolism. Elevated levels of intracellular calcium have been shown to increase the expression of myonectin in skeletal muscle [120,121]. Myonectin is elevated in adults with T2D and increased adiposity, relative to healthy individuals, likely as a compensatory mechanism against insulin resistance [122]. However, diet-induced obesity in mice does not cause this compensatory mechanism—the muscle mRNA levels and circulating protein levels of myonectin were reduced relative to control mice, and subsequent voluntary exercise increased myonectin gene expression and circulating protein levels [123]. This conundrum was resolved when it was determined that myonectin levels are raised after feeding, indicating that myonectin secretion could be regulated by substrate availability. For example, overnight fasting decreases myonectin levels, and subsequent feeding with glucose or emulsified lipids increases circulating myonectin levels in mouse models [123]. Overall, myonectin is an important mediator in inter-organ cross-talk and its secretion by skeletal muscle increases with the higher availability of glucose and fatty acids in the insulin-resistant and T2D state as a compensatory mechanism to improve glucose tolerance and increase fatty acid oxidation [122,124].

**Myostatin:** Myostatin, also named growth and differentiation factor-8 (GDF-8), is expressed in both embryonic and adult skeletal muscle. It is secreted by skeletal muscle and cardiac cells and is reported to inhibit muscle growth and differentiation and reduce skeletal muscle mass [125,126]. Consistent with this, myostatin-suppressed mice and cattle are larger than control animals, suggesting that myostatin functions as a ‘brake’ to suppress skeletal muscle growth [127,128]; similar findings have been reported for humans and dogs [129]. Myostatin is a member of the transforming growth factor β (TGFβ) superfamily. Mechanistically, myostatin binds to activin type IIA and IIB receptors (ActRIIA/B) and TGFβ receptors (TGFβRII) at the plasma membrane. The myostatin-mediated muscle growth impairment is caused by activating activin, which in turn phosphorylates SMAD2/3 and promotes the establishment of a heterotrimeric complex with SMAD4 [130]. Furthermore, the inhibition of myostatin-induced reactive oxygen species (ROS) is an effective treatment for reducing muscle wasting during sarcopenia [131]. Interestingly, myostatin ablation in mice skeletal muscle was also discovered to prevent fat mass gain [132]. While myostatin was initially discovered as a myokine, it was later determined to also be secreted by adipose tissue, and thus is termed as an adipo-myokine [133]. Consistent with a role in adipose tissue, myostatin knockout mice had shown a reduced fat pad mass and were resistant to obesity and insulin resistance [134,135,136]. Further, the inhibition of myostatin, via a loss-of-function mutation in one or both alleles of the *myostatin* gene, improves whole-body insulin sensitivity and alleviates the development of insulin resistance in obese mice; the genetic loss of myostatin also improves insulin sensitivity and glucose tolerance in severely obese mouse models [135,137,138]. Moreover, the muscle-specific inhibition of myostatin increases the protein levels of GLUT1 and GLUT4 in rat muscle [139], providing a mechanistic basis for the beneficial effects of myostatin inhibition to improve glucose tolerance. Together, myostatin is a negative myokine/adipokine that impairs glucose uptake, enhances adiposity, and impairs muscle growth and function.

## 3. Muscle-to-Pancreas Cross-Talk

**Figure 2:**
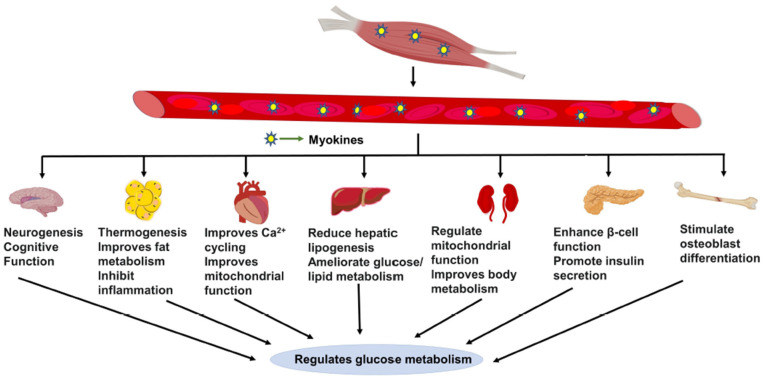
Myokine-mediated systemic regulation. Skeletal muscle-secreted myokines are involved in cross-talk with other internal organs to regulate cognitive function, stimulate osteoblast differentiation, enhance islet β-cell function, promote insulin secretion, regulate mitochondrial function, increase lipolysis and promote glucose oxidation. Through their role in organ cross-talk and systemic regulation of energy metabolism, myokines hold substantial promise for reducing inflammation and reducing the risk of insulin resistance and type 2 diabetes. This figure was created with Biorender.com.

Skeletal muscle influences insulin secretion by interacting with the pancreas through humoral factors [46,140,141,142]. The discovery that skeletal muscle has an endocrine function has provided key insights into inter-organ cross-talk (Figure 2). For example, skeletal muscle-specific PGC1α knockout mice displayed impaired insulin secretion [141]. Additionally, the skeletal muscle-specific enrichment of RING-finger protein 1 (MuRF1) modifies the muscle metabolism, which stimulates pancreatic insulin secretion [142]. In addition, conditioned media from human muscle cells enriched with IL-6, IL8/CXCL8, MCP1/CCL2, fractalkine/CX3CL1, and RANTES/CCL5 increased glucose-stimulated insulin secretion (GSIS) from rat and human primary β-cells [46,143,144]. β-cell responsiveness to myokines is linked to the presence of many of the myokine receptors on the islet β-cells. Of note, the overexpression of the p21–activated kinase 1 (PAK1, required for the non-canonical insulin-stimulated GLUT4 vesicle translocation in skeletal muscle cells) [145,146,147] in rat L6 myoblasts or myotubes releases into the conditioned media muscle-derived circulating factor(s) that are capable of enhancing β-cell function [148]. Interestingly, myokines secreted into conditioned media from human T2D skeletal muscle cells cultured under diabetogenic conditions were shown to suppress GSIS from β-cells [149]. β-cell responsiveness to myokines is linked to the presence of many of the myokine receptors on the islet β-cells. Myokines released from insulin-sensitive or insulin-resistant skeletal muscle that have positive and/or negative effects on the function and survival β-cells are discussed in this section.

**Chemokine C-X-C motif ligand 10:** Chemokine C-X-C motif ligand 10 (CXCL10), also called IFNγ-induced protein 10 (IP-10), is a protein produced and secreted by several cell types, including skeletal muscle. It is known as an inflammatory *chemokine* that exhibits pleiotropic effects on a wide range of pathophysiological processes, including T2D. Cultured insulin-resistant skeletal muscle cells secrete higher levels of CXCL10 than control cells [150], indicating that CXCL10 may have detrimental functions, and CXCL10 is increased in serum of T2D individuals relative to healthy individuals [151,152,153,154]. Direct CXCL10 treatment of pancreatic β-cells induces β-cell apoptosis. Consistent with this, the provision of conditioned media from insulin-resistant human myotubes, which express elevated levels of CXCL10, resulted in β-cell apoptosis [46].

**Follistatin:** Follistatin is vital for the formation and growth of skeletal muscle fibers [155,156], and it has autocrine and paracrine functions in metabolism [157,158]. In response to physical activity, follistatin levels rapidly increase. Follistatin is then secreted into the bloodstream where, in a paracrine function, it targets the pancreas [159]. Acute follistatin treatment reduces glucagon secretion from the pancreatic α-cells. Conversely, chronic follistatin treatment prevents apoptosis and induces the proliferation of rat β-cells [160]. Follistatin acts as an antagonist of activin A, thereby suppressing the effects of activin A on SMAD2/3, relieving the activin A-mediated suppression of skeletal muscle glucose uptake, and alleviating the transcriptional repression of canonical β-cell transcription factors including MafA and Pdx1 [161,162]. Counterintuitively, patients with T2D show elevated levels of follistatin, yet it remains unclear whether this is a consequence of long-term defects in glucose metabolism [157]. Further studies are required to understand the extent to which follistatin acts as an autocrine myokine to positively impact pancreatic function.

**Irisin:** As described previously, Irisin acts on the skeletal muscle to increase glucose oxidation and reduce circulatory glucose levels [64,163]. In addition to its autocrine role in muscle-to-muscle communication, Irisin also improves the proliferation of INS-1E β-cells, increases their insulin production, and protects them from hyperglycemia-induced apoptosis [164]. Furthermore, the administration of Irisin to T2D rats (diabetes induced via HFD feeding plus streptozotocin treatment) led to an improved glucose tolerance along with lowered fasting blood glucose [164]. Similarly, mice administered Irisin also showed improved glucose-stimulated insulin secretion as well as increased β-cell proliferation in vivo, suggesting that Irisin plays a positive role in pancreatic β-cells. Indeed, later ex vivo studies supported this idea as mouse and human islets cultured with muscle-derived Irisin-enriched conditioned media from palmitate-treated L6 myotubes displayed increased insulin biosynthesis and protection from palmitate-induced β-cell apoptosis [165]. Therefore, Irisin could be considered as the positive myokine that regulates energy metabolism both via improved skeletal muscle insulin sensitivity and islet β-cell insulin secretion.

**Fractalkine:** Known also as Chemokine (C-X3-C motif) ligand 1 (CX3CL1), Fractalkine is a myokine with a potentially beneficial function in muscle injury and repair [166]. Consistent with this concept, the expression of Fractaline is increased in insulin-resistant human skeletal muscle cells ex vivo [46], and Fractaline treatment of islets ex vivo led to elevated intracellular calcium (Ca^2+^) and triggered insulin secretion in both mouse and human islets [167]. Furthermore, the chronic administration of a Fractaline analog in various rodent models of obesity improved glucose tolerance, increased β-cell glucose-stimulated insulin secretion, and reduced β-cell apoptosis, highlighting its positive effect in regulating glucose homeostasis [167,168]. Fractaline treatment also prevents TNFα-induced dysfunction in primary β-cells [169]. Due to its positive functions in muscle-to-pancreas cross-talk, Fractaline may be of great interest as a new therapeutic agent for T2D.

## 4. Myokine Cross-Talk with Other Major Metabolic Organs

In addition to autocrine actions and cross-talk with the pancreas, myokines mediate muscle-to-organ cross-talk with the brain, adipose tissue, heart, kidney, bone, gut, liver, vascular bed, and skin (Table 1). For example, in adipose tissue, myokines play a central role in energy metabolism, the regulation of lipid mobilization, and glucose oxidation. Thus, given that T2D is a disease of organs beyond just muscle and the pancreas, understanding the additional muscle-to-organ interactions may contribute to the development of effective therapeutic strategies to prevent or reverse metabolic disorders including T2D.

### 4.1. Muscle-to-Adipose Tissue Cross-Talk

Exercise-induced myokines regulate lipid metabolism, induce the formation of brown adipose tissue, and inhibit inflammatory responses. Numerous studies indicate that skeletal muscle-derived myokines modulate the pathophysiological functions of adipose tissue. For instance, the circulating levels of IL-6 are increased during muscle contraction and regulate metabolic actions in adipose tissue. IL-6 secretion is mediated through increasing cytosolic Ca^2+^ and activating P38 mitogen-activated protein kinase or calcineurin. In line with this, IL-6 is predominantly secreted by slow-twitch fibers; circulating IL-6 induces the expression of brown adipose tissue-associated uncoupling protein 1 (UCP1) in white adipose tissue as a response to cold adaptation and participates in fat browning [199]. While recombinant human IL-6 (rhIL-6) treatment in humans increased fatty acid oxidation, it had no effect on glucose metabolism [95]. Further, the ex vivo treatment of rodent epididymal adipose tissue with IL-6 enhanced lipolysis [200], and humans infused with IL-6 exhibited increased whole-body lipolysis and fat oxidation [95,201]. Since IL-6 activation in subcutaneous adipose tissue may induce leptin-mediated GLP-1 release [202], and GLP-1 potentiates glucose-stimulated insulin release, selective IL-6 activation could be a beneficial strategy to prevent the development of T2D in insulin-resistant patients.

Meteorin-like (Metrnl) is a novel muscle-derived factor reported to regulate energy homeostasis. Exercise induces Metrnl expression in muscle, and it is released into the circulation where it exerts anti-inflammatory effects on the adipose tissue macrophages of HFD-fed mice by suppressing NLRP3 inflammasome activation in subcutaneous and visceral adipose tissue [203]. Exercise-induced circulating Metrnl also enhances energy expenditure, increases anti-inflammatory cytokines, and activates beige fat thermogenesis in mice [204]. In addition, follistatin enrichment in mice decreases abdominal fat content, increases glucose clearance, and improves plasma lipid profiles via enhancing AMPK-mediated energy expenditure [205]. In addition, follistatin induces adipocyte differentiation and regulates energy metabolism in cultured primary mouse embryonic fibroblasts [206]. Finally, Irisin mediates white adipose tissue browning and ameliorates perivascular adipose tissue dysfunction in HFD-induced obese mice [64,207]. Recombinant FNDC5 (Irisin precursor) treatment of primary subcutaneous adipocytes increased the expression of brown adipose tissue genes including *UCP1*, *Elovl3*, *Cox7a*, and *Otop1*, and increased the mitochondrial content, oxygen consumption, and a beige phenotype [208]. Recombinant Irisin treatment of 3T3-L1 mouse and rat primary adipocytes similarly increased the mRNA levels of brown adipose tissue-specific genes, which was regulated via the p38 MAPK and ERK signaling pathways [72]. Overall, these findings demonstrate that muscle-derived myokines play central roles in the regulation of fat browning, thermogenesis, and lipolysis, and these regulatory properties may help to protect/treat metabolic disorders and obesity.

### 4.2. Muscle-to-Brain Cross-Talk

Recent research suggests that myokines are involved in muscle–brain communication. Reduced BDNF is associated with T2D, coronary disorders, and atherosclerosis in humans [209,210]. BDNF is a fasting-induced myokine that controls the metabolic reprograming of lipid and glucose oxidation for ATP production during metabolic stress [90], and specifically, muscle-derived BDNF facilitates metabolic adaption during nutrient insufficiency in a female-specific manner; deficient BDNF production in skeletal muscle promotes the development of metabolic myopathies and insulin resistance. Furthermore, a peripheral injection of BDNF reduces hyperglycemia in obese rodents, [211,212]. Consistent with this, BDNF knockout mice develop mature-onset obesity with elevated levels of serum leptin, insulin, glucose, and cholesterol, and an increased body mass index [213,214].

Cathepsin B (CTSB) is an exercise-induced myokine required for adult neurogenesis and memory improvement. Running and treadmill training in animals and humans increases plasma CSTB levels, which can cross the blood–brain barrier and induce BDNF secretion in mice [215]. In addition, conditioned media from L6 myotubes treated with the AMPK agonist 5-aminoimidazole-4-carboxamide ribonucleotide (AICAR), to model the effects of exercise in vitro, showed elevated CSTB levels. The brain is well known to be the major metabolic consumer of glucose, thus exercise-mediated myokines that improve brain functions, such as appetite control and sleep cycles, to enhance glucose oxidation can regulate whole-body energy metabolism. Despite the referenced findings, additional studies are essential to better understand the muscle-to-brain cross-talk in the context of peripheral insulin resistance/sensitivity and energy regulation.

### 4.3. Muscle-to-Liver Cross-Talk

The liver is the central hub for metabolism; it maintains a constant energy supply to other organs via regulating various pathways including glycogenesis, glycogenolysis, gluconeogenesis, and lipolysis. Exercise-mediated increases in energy demand are compensated for by increased hepatic glucose production. Myokines such as IL-6, Irisin, BAIBA, myonectin, and FGF21 are involved in the control of metabolic events in the liver and regulate systemic energy homeostasis. For example, human muscle-derived IL-6 signals to hepatic cells to produce glucose during exercise [216]. In the absence of exercise, however, IL-6 infusion in lean and obese mice enhanced the AKT-mediated downregulation of liver glucogenesis [217], demonstrating that IL-6 can serve as a signaling regulator that can either increase or decrease liver glucose production based on the body’s energy demand. IL-6 can be upregulated in the gastrocnemius and liver in mice by the delivery of the pro-inflammatory cytokine interleukin-17 (IL-17), which correlates with inflammatory induction [218]. IL-17 induction in adult rats via an acute single bout of strenuous exercise also correlated with increased inflammation of skeletal muscles [219]. Furthermore, obesity-induced IL-17 is considered central to the development and progression of non-alcoholic fatty liver disease to steatohepatitis [220]; increased peripheral IL-17 levels are associated with early atherosclerosis in obese patients [221,222].

In other examples, Irisin, BAIBA, myonectin and FGF21 impact liver. Irisin content in circulation is negatively correlated with circulatory high-density lipoprotein, cholesterol, and intrahepatic triglyceride content, protecting against fatty liver [214]. Indeed, sarcopenic patients with liver cirrhosis showed decreased serum Irisin concentrations [223]. Furthermore, studies of non-alcoholic fatty liver disease patients have revealed that Irisin levels are low in patients with moderate-to-severe steatosis [224]. Mechanistically, Irisin inhibits hepatic gluconeogenesis and increases glycogen synthesis mediated by the PI3K/Akt pathway in T2D mice and HepG2 cells [225]. BAIBA, released from the muscle after exercise, increases hepatic β-oxidation [83]. Myonectin increases fatty acid uptake into cultured hepatocytes via a mechanism involving the upregulation of genes involved in fatty acid utilization, including *CD36*, *FATP1*, *FABP1*, and *FABP4* [123]. Lastly, chronic treatment with FGF21 in the db/db diabetic mouse model improved hepatic glucose uptake and suppressed hepatic gluconeogenesis-mediated glucose release [226]. Taken together, these findings demonstrate that the liver is a target organ for myokine action, which regulates its metabolic function in response to the skeletal muscle’s energy requirements.

### 4.4. Muscle-to-Heart/Kidney/Bone Tissue Cross-Talk

Skeletal muscle myokines also mediate communication with the heart, bone, blood and kidney to regulate metabolic functions [227,228,229,230].

*Muscle-Heart cross-talk:* Myokines such as apelin, myonectin, Irisin, and BDNF decrease the risk of cardiovascular complications in sarcopenia patients [231]. Indeed, myonectin heterozygous knockout mice subjected to an ischemia-reperfusion injury exhibited increases in myocardial infarct size, apoptosis, cardiac dysfunction, and pro-inflammatory gene levels compared with the wild-type. In contrast, mice with skeletal muscle-specific overexpression of myonectin showed reduced myocardial damage after ischemia-reperfusion [232].

*Muscle-Kidney cross-talk:* Primary kidney tubule cells cultured with serum enriched in Irisin, BDNF, and IL-15 showed increased levels of maximal respiratory capacity and ATP-coupled respiration [233]. This study further showed that recombinant Irisin counteracts TGF-β1-induced pathological metabolic reprogramming in primary kidney tubule cells, which improves kidney function and blocks fibrosis.

*Muscle-Bone cross-talk*: Irisin treatment in young male C57BL/6 mice correlated with increased cortical bone mass, geometry and strength [234]. As described in previous sections, IL-6 can exert positive or negative actions, and in bone, the effects are largely negative. For example, IL-6 promotes osteoclastogenesis by inducing the release of Receptor Activator of Nuclear factor Kappa-Β (RANK) from osteoblasts, osteocytes, and leukocytes. RANK promotes the expression of its specific ligand (RANKL) by osteoclasts, leading to a net resorption of bone [235]. In addition, myostatin, activin, IL-6 and CNTF negatively impact bone growth and function, whereas myokines such as IGF-1, FGF-2, IL-7, IL-15, Follistatin and osteonectin exert positive actions on bone function [236].

## 5. Perspectives: Myokines as Therapeutic Targets for T2D

Leveraging the positive actions of myokines on insulin secretion, insulin sensitivity, and energy metabolism could lead to important novel therapies for T2D. As expected, there has been a surge in research on contraction-regulated myokines that promote β-cell mass and function [237,238]. Beneficial myokines, including Irisin, fractalkine, FGF21, myonectin, and IL-15, improve β-cell mass and/or function, which regulates glucose and lipid metabolism. For example, FGF21-boosted insulin secretion is accompanied by decreased triacylglycerol levels and increased insulin sensitivity via skeletal muscle glucose uptake, white adipose tissue lipolysis and browning, and increased energy expenditure [239,240]. Similarly, human obese/overweight women participating in an aerobic exercise program were found to have higher serum myonectin levels, with significantly decreased susceptibility for insulin resistance [124]. In line with this, Chinese T2D patients displayed decreased serum myonectin levels [241], suggesting that myonectin may serve as a rheostat for insulin sensitivity. In another approach, antagonists of ‘detrimental myokines’ could provide a therapeutic benefit for metabolic disease. One example of this is the antagonism of myostatin, which was used to prevent/reverse muscle wasting [242], but could be extrapolated further to metabolic disease applications. These examples together demonstrate the potential clinical importance of targeting myokines for metabolic disease. However, there are points of controversy regarding myokine targeting in humans. An example of disparate findings is shown with the myokine IL-6, where some report that IL-6 increases β-cell function [140,238] and others have demonstrated no significant changes in insulin secretion in response to physiological levels of IL-6 [243]. In addition, elevated IL-6 and IL-17 were detected in elderly patients with sarcopenia and an impaired metabolism, as compared with non-sarcopenic elderly persons [244]. Irisin is another recently discovered contraction-regulated myokine that induces subcutaneous white adipose tissue browning, thereby enhancing whole-body energy expenditure, but this mechanism is restricted to rodents and primates to date [245,246]. Additionally, the Irisin treatment of human primary pre-adipocytes did not induce a shift of white to brown adipose tissue [245], thus calling into question the application of Irisin as a therapeutic agent [241].

FGF21 mimetics and analogues have advanced to clinical trials in patients with T2D, obesity and non-alcoholic steatohepatitis [247]. Daily subcutaneous administration of the LY2405319-human FGF21 analogue for 28 days in obese and T2D patients (NCT01869959) resulted in reduced mean fasting insulin levels, suggesting potential improvement in insulin sensitivity, although direct measures of insulin action were not evaluated in this study [60]. Nevertheless, the trial provided information regarding the drug safety, tolerability, and pharmacokinetic/pharmacodynamics of LY2405319, sufficient to support a 28-day phase 1b proof-of-concept trial. Another trial tested PF-05231023, a stable long-acting human-FGF21 analogue (NCT01396187), which reported decreased body weight, but did not reduce plasma insulin concentrations nor alter glycemia [61]. A third trial, a phase 1 multiple ascending dose study in individuals with T2D (NCT01856881), tested AKR-001, an Fc-FGF21 analog harboring stabilized N- and C-terminal domains of FGF21, resulting in improved serum markers for insulin sensitivity and dyslipidemia, and demonstrated trends toward improvements in glycemic control through enhancing insulin sensitivity under fasting and fed conditions [248]. Based on these findings, compounds that target FGF21 are emerging that carry therapeutic promise.

In summary, the mechanisms of myokine actions on β-cell viability and function are mostly validated in rodent models, and only a few so far have a demonstrated mechanism in humans. As with many therapeutic targets, the myokine levels in healthy and diseased individuals vary significantly, leading to differential outcomes. In addition, the myokine receptor abundance is often not quantified, and could be a contributing factor to the differential outcomes observed thus far (i.e., the muscle may increase the release of the myokine into circulation, but a paucity of target cell receptors for the myokine could preclude its cellular action and allow T2D to persist). Nevertheless, the list of new myokines is steadily increasing, and comprehensive analyses of myokine networks, local and systemic levels in health and disease, and their synergistic functions, will help to determine druggable targets in the near future.

## Figures and Tables

**Table 1:** Myokines involved in organ cross-talk and regulation of metabolism.

Myokines	Organ Cross-Talk	Role in Energy Metabolism
Adiponectin	Adipose tissue, Pancreas	↑ Glucose metabolism [170,171]
Apelin	Heart, Pancreas	↑Insulin sensitivity [172] ↑Glucose uptake [173,174] ↑ β-oxidation [175]
BAIBA	Fat, Liver, Bone	↑ Mitochondrial metabolism [81,83] ↑ Insulin sensitivity [82]
CX3CL1/Fractaline	Pancreas	↑ Fatty acid oxidation [176]
FGF21	Adipose tissue, Liver	↑ Insulin sensitivity [59,177] ↑ Lipolysis [178,179] ↑ Oxidative capacities [180] ↓ Triglycerides l [181,182]
IL-15	Adipose tissue, Bone	↑ Glucose uptake [183] ↑ Fatty acid oxidation [115] ↑ Mitochondrial function [183] ↓Oxidative stress and lipid accumulation [183]
IL-6	Liver, Adipose tissue, Pancreas, Bone	↑ Insulin sensitivity [184] ↑ Glucose uptake [18,184,185] ↑ Fatty acid oxidation [186] ↑ Glycogen synthesis [186]
IL-10	Adipose tissue	↑ Glucose metabolism [187,188]
Irisin	Adipose tissue, Brain, Bone, Heart, Blood, Kidney	↑Glucose uptake [64,189] ↑ β-oxidation and mitochondrial biogenesis [63,189]
METRNL	Adipose tissue	↑ Glucose metabolism [190,191]
Musclin (osteocrin)	Heart, Bone, Brain	↓Decrease glucose uptake and insulin sensitivity
Myonectin	Heart, Liver, Adipocytes	↑ Glucose uptake [120] ↑ β-oxidation [123,192]
Myostatin	Adipose tissue, Liver, Bone, Muscle	↓Decrease glucose uptake and insulin sensitivity [193,194,195]
Osteoglycin	Muscle, Bone	↑ Glucose metabolism [196] ↑ Fatty acid oxidation [197]
SPARC	Adipose tissue, Muscle	↑ Glucose tolerance [76] inhibits adipogenesis [198]

## Data Availability

Disclosure: D.C.T. is a scientific consultant for True Binding, Inc.

## References

[1] Fox C.S., Golden S.H., Anderson C., Bray G.A., Burke L.E., de Boer I.H., Deedwania P., Eckel R.H., Ershow A.G., Fradkin J. (2015). Update on Prevention of Cardiovascular Disease in Adults with Type 2 Diabetes Mellitus in Light of Recent Evidence. Circulation.

[2] Ariza L., Pages G., García-Lareu B., Cobianchi S., Otaegui P., Ruberte J., Chillon M., Navarro X., Bosch A. (2014). Experimental diabetes in neonatal mice induces early peripheral sensorimotor neuropathy. Neuroscience.

[3] Yang H., Xie T., Li D., Du X., Wang T., Li C., Song X., Xu L., Yi F., Liang X. (2019). Tim-3 aggravates podocyte injury in diabetic nephropathy by promoting macrophage activation via the NF-κB/TNF-α pathway. Mol. Metab..

[4] Saadane A., Lessieur E.M., Du Y., Liu H., Kern T.S. (2020). Successful induction of diabetes in mice demonstrates no gender difference in development of early diabetic retinopathy. PLoS ONE.

[5] Thyfault J.P., Bergouignan A. (2020). Exercise and metabolic health: Beyond skeletal muscle. Diabetologia.

[6] Ferrannini E., Simonson D.C., Katz L.D., Reichard G., Bevilacqua S., Barrett E.J., Olsson M., DeFronzo R.A. (1988). The disposal of an oral glucose load in patients with non-insulin-dependent diabetes. Metabolism.

[7] Centers for Disease Control and Prevention (2017). National Diabetes Statistics Report.

[8] Sun H., Saeedi P., Karuranga S., Pinkepank M., Ogurtsova K., Duncan B.B., Stein C., Basit A., Chan J.C., Mbanya J.C. (2022). IDF Diabetes Atlas: Global, regional and country-level diabetes prevalence estimates for 2021 and projections for 2045. Diabetes Res. Clin. Pr..

[9] Centers for Disease Control and Prevention National Diabetes Statistics Report. https://www.cdc.gov/diabetes/data/statistics-report/index.html.

[10] Bagnasco A., Di Giacomo P., Mora R.D.R.D., Catania G., Turci C., Rocco G., Sasso L. (2014). Factors influencing self-management in patients with type 2 diabetes: A quantitative systematic review protocol. J. Adv. Nurs..

[11] Marín-Peñalver J.J., Martín-Timón I., Sevillano-Collantes C., Del Cañizo-Gómez F.J. (2016). Update on the treatment of type 2 diabetes mellitus. World J. Diabetes.

[12] Pot G.K., Battjes-Fries M.C., Patijn O.N., van der Zijl N., Pijl H., Voshol P. (2020). Lifestyle medicine for type 2 diabetes: Practice-based evidence for long-term efficacy of a multicomponent lifestyle intervention (Reverse Diabetes2 Now). BMJ Nutr. Prev. Health.

[13] Chaudhury A., Duvoor C., Reddy Dendi V.S., Kraleti S., Chada A., Ravilla R., Marco A., Shekhawat N.S., Montales M.T., Kuriakose K. (2017). Clinical Review of Antidiabetic Drugs: Implications for Type 2 Diabetes Mellitus Management. Front. Endocrinol..

[14] Phung O.J., Scholle J.M., Talwar M., Coleman C. (2010). Effect of Noninsulin Antidiabetic Drugs Added to Metformin Therapy on Glycemic Control, Weight Gain, and Hypoglycemia in Type 2 Diabetes. JAMA.

[15] Carson B.P. (2017). The Potential Role of Contraction-Induced Myokines in the Regulation of Metabolic Function for the Prevention and Treatment of Type 2 Diabetes. Front. Endocrinol..

[16] Pedersen B.K. (2019). Physical activity and muscle–brain crosstalk. Nat. Rev. Endocrinol..

[17] Pedersen B.K., Febbraio M.A. (2012). Muscles, exercise and obesity: Skeletal muscle as a secretory organ. Nat. Rev. Endocrinol..

[18] Carey A.L., Steinberg G.R., Macaulay S.L., Thomas W.G., Holmes A.G., Ramm G., Prelovsek O., Hohnen-Behrens C., Watt M.J., James D.E. (2006). Interleukin-6 Increases Insulin-Stimulated Glucose Disposal in Humans and Glucose Uptake and Fatty Acid Oxidation In Vitro via AMP-Activated Protein Kinase. Diabetes.

[19] Serrano A.L., Baeza-Raja B., Perdiguero E., Jardí M., Muñoz-Cánoves P. (2008). Interleukin-6 Is an Essential Regulator of Satellite Cell-Mediated Skeletal Muscle Hypertrophy. Cell Metab..

[20] Toth K.G., McKay B.R., De Lisio M., Little J.P., Tarnopolsky M.A., Parise G. (2011). IL-6 Induced STAT3 Signalling Is Associated with the Proliferation of Human Muscle Satellite Cells Following Acute Muscle Damage. PLoS ONE.

[21] Pedersen B.K. (2013). Muscle as a Secretory Organ. Compr. Physiol..

[22] Pedersen B.K., Åkerström T.C., Nielsen A.R., Fischer C.P. (2007). Role of myokines in exercise and metabolism. J. Appl. Physiol..

[23] Huh J.Y. (2018). The role of exercise-induced myokines in regulating metabolism. Arch. Pharmacal. Res..

[24] Yamanaka M., Itakura Y., Inoue T., Tsuchida A., Nakagawa T., Noguchi H., Taiji M. (2006). Protective effect of brain-derived neurotrophic factor on pancreatic islets in obese diabetic mice. Metabolism.

[25] Aoi W., Naito Y., Takagi T., Tanimura Y., Takanami Y., Kawai Y., Sakuma K., Hang L.P., Mizushima K., Hirai Y. (2013). A novel myokine, secreted protein acidic and rich in cysteine (SPARC), suppresses colon tumorigenesis via regular exercise. Gut.

[26] Whitham M., Febbraio M.A. (2016). The ever-expanding myokinome: Discovery challenges and therapeutic implications. Nat. Rev. Drug Discov..

[27] Raschke S., Eckardt K., Holven K.B., Jensen J., Eckel J. (2013). Identification and Validation of Novel Contraction-Regulated Myokines Released from Primary Human Skeletal Muscle Cells. PLoS ONE.

[28] Hartwig S., Raschke S., Knebel B., Scheler M., Irmler M., Passlack W., Muller S., Hanisch F.-G., Franz T., Li X. (2014). Secretome profiling of primary human skeletal muscle cells. Biochim. Biophys. Acta (BBA)-Proteins Proteom..

[29] Norheim F., Raastad T., Thiede B., Rustan A.C., Drevon C.A., Haugen F. (2011). Proteomic identification of secreted proteins from human skeletal muscle cells and expression in response to strength training. Am. J. Physiol. Metab..

[30] Chan X.C.Y., McDermott J.C., Siu K.W.M. (2007). Identification of Secreted Proteins during Skeletal Muscle Development. J. Proteome Res..

[31] Chan C.Y.X., Masui O., Krakovska O., Belozerov V.E., Voisin S., Ghanny S., Chen J., Moyez D., Zhu P., Evans K.R. (2011). Identification of Differentially Regulated Secretome Components During Skeletal Myogenesis. Mol. Cell. Proteom..

[32] Severinsen M.C.K., Pedersen B.K. (2020). Muscle–Organ Crosstalk: The Emerging Roles of Myokines. Endocr. Rev..

[33] Febbraio M.A., Pedersen B.K. (2020). Who would have thought—Myokines two decades on. Nat. Rev. Endocrinol..

[34] Guo A., Li K., Xiao Q. (2020). Sarcopenic obesity: Myokines as potential diagnostic biomarkers and therapeutic targets?. Exp. Gerontol..

[35] Crane J., MacNeil L.G., Lally J.S., Ford R.J., Bujak A.L., Brar I.K., Kemp B., Raha S., Steinberg G., Tarnopolsky M.A. (2015). Exercise-stimulated interleukin-15 is controlled by AMPK and regulates skin metabolism and aging. Aging Cell.

[36] Vinel C., Lukjanenko L., Batut A., Deleruyelle S., Pradère J.-P., Le Gonidec S., Dortignac A., Geoffre N., Pereira O., Karaz S. (2018). The exerkine apelin reverses age-associated sarcopenia. Nat. Med..

[37] Vuillermoz B., Wegrowski Y., Contet-Audonneau J.-L., Danoux L., Pauly G., Maquart F.-X. (2005). Influence of aging on glycosaminoglycans and small leucine-rich proteoglycans production by skin fibroblasts. Mol. Cell. Biochem..

[38] Kucera R., Topolcan O., Pecen L., Kinkorova J., Svobodova S., Windrichová J., Fuchsova R. (2015). Reference values of IGF1, IGFBP3 and IGF1/IGFBP3 ratio in adult population in the Czech Republic. Clin. Chim. Acta.

[39] Li L., Yang G., Li Q., Tang Y., Yang M., Yang H., Li K. (2006). Changes and Relations of Circulating Visfatin, Apelin, and Resistin Levels in Normal, Impaired Glucose Tolerance, and Type 2 Diabetic Subjects. Exp. Clin. Endocrinol. Diabetes.

[40] Quinn L.S., Anderson B.G., Strait-Bodey L., Wolden-Hanson T. (2010). Serum and muscle interleukin-15 levels decrease in aging mice: Correlation with declines in soluble interleukin-15 receptor alpha expression. Exp. Gerontol..

[41] Hulmi J.J., Silvennoinen M., Lehti M., Kivelä R., Kainulainen H. (2012). Altered REDD1, myostatin, and Akt/mTOR/FoxO/MAPK signaling in streptozotocin-induced diabetic muscle atrophy. Am. J. Physiol. Metab..

[42] Efthymiadou A., Vasilakis I.-A., Giannakopoulos A., Chrysis D. (2021). Myostatin serum levels in children with type 1 diabetes mellitus. Hormones.

[43] Wang F., Liao Y., Li X., Ren C., Cheng C., Ren Y. (2012). Increased circulating myostatin in patients with type 2 diabetes mellitus. J. Huazhong Univ. Sci. Technol..

[44] Kwon J., Moon K., Min K.-W. (2020). Exercise-Induced Myokines can Explain the Importance of Physical Activity in the Elderly: An Overview. Healthcare.

[45] Park K., Ahn C.W., Park J.S., Kim Y., Nam J.S. (2020). Circulating myokine levels in different stages of glucose intolerance. Medicine.

[46] Bouzakri K., Plomgaard P., Berney T., Donath M.Y., Pedersen B.K., Halban P.A. (2011). Bimodal Effect on Pancreatic β-Cells of Secretory Products from Normal or Insulin-Resistant Human Skeletal Muscle. Diabetes.

[47] Ciaraldi T.P., Ryan A.J., Mudaliar S.R., Henry R.R. (2016). Altered Myokine Secretion Is an Intrinsic Property of Skeletal Muscle in Type 2 Diabetes. PLoS ONE.

[48] Fisher F.M., Maratos-Flier E. (2016). Understanding the Physiology of FGF21. Annu. Rev. Physiol..

[49] Kuro-o M. (2018). Ageing-related receptors resolved. Nature.

[50] Lee S., Choi J., Mohanty J., Sousa L.P., Tome F., Pardon E., Steyaert J., Lemmon M., Lax I., Schlessinger J. (2018). Structures of β-klotho reveal a ‘zip code’-like mechanism for endocrine FGF signalling. Nature.

[51] Adams A.C., Yang C., Coskun T., Cheng C.C., Gimeno R.E., Luo Y., Kharitonenkov A. (2012). The breadth of FGF21’s metabolic actions are governed by FGFR1 in adipose tissue. Mol. Metab..

[52] Kolumam G., Chen M.Z., Tong R., Zavala-Solorio J., Kates L., van Bruggen N., Ross J., Wyatt S.K., Gandham V.D., Carano R.A. (2015). Sustained Brown Fat Stimulation and Insulin Sensitization by a Humanized Bispecific Antibody Agonist for Fibroblast Growth Factor Receptor 1/βKlotho Complex. EBioMedicine.

[53] Lan T., Morgan D.A., Rahmouni K., Sonoda J., Fu X., Burgess S.C., Holland W.L., Kliewer S.A., Mangelsdorf D.J. (2017). FGF19, FGF21, and an FGFR1/β-Klotho-Activating Antibody Act on the Nervous System to Regulate Body Weight and Glycemia. Cell Metab..

[54] Hojman P., Pedersen M., Nielsen A.R., Krogh-Madsen R., Yfanti C., Åkerstrom T., Nielsen S., Pedersen B.K. (2009). Fibroblast Growth Factor-21 Is Induced in Human Skeletal Muscles by Hyperinsulinemia. Diabetes.

[55] Izumiya Y., Bina H.A., Ouchi N., Akasaki Y., Kharitonenkov A., Walsh K. (2008). FGF21 is an Akt-regulated myokine. FEBS Lett..

[56] Coskun T., Bina H.A., Schneider M.A., Dunbar J.D., Hu C.C., Chen Y., Moller D.E., Kharitonenkov A. (2008). Fibroblast Growth Factor 21 Corrects Obesity in Mice. Endocrinology.

[57] Xu J., Lloyd D.J., Hale C., Stanislaus S., Chen M., Sivits G., Vonderfecht S., Hecht R., Li Y.-S., Lindberg R.A. (2009). Fibroblast Growth Factor 21 Reverses Hepatic Steatosis, Increases Energy Expenditure, and Improves Insulin Sensitivity in Diet-Induced Obese Mice. Diabetes.

[58] Kharitonenkov A., Wroblewski V.J., Koester A., Chen Y.-F., Clutinger C.K., Tigno X.T., Hansen B.C., Shanafelt A.B., Etgen G.J. (2007). The Metabolic State of Diabetic Monkeys Is Regulated by Fibroblast Growth Factor-21. Endocrinology.

[59] Foltz I.N., Hu S., King C., Wu X., Yang C., Wang W., Weiszmann J., Stevens J., Chen J.S., Nuanmanee N. (2012). Treating Diabetes and Obesity with an FGF21-Mimetic Antibody Activating the βKlotho/FGFR1c Receptor Complex. Sci. Transl. Med..

[60] Gaich G., Chien J.Y., Fu H., Glass L.C., Deeg M.A., Holland W.L., Kharitonenkov A., Bumol T., Schilske H.K., Moller D.E. (2013). The Effects of LY2405319, an FGF21 Analog, in Obese Human Subjects with Type 2 Diabetes. Cell Metab..

[61] Talukdar S., Zhou Y., Li D., Rossulek M., Dong J., Somayaji V., Weng Y., Clark R., Lanba A., Owen B.M. (2016). A Long-Acting FGF21 Molecule, PF-05231023, Decreases Body Weight and Improves Lipid Profile in Non-human Primates and Type 2 Diabetic Subjects. Cell Metab..

[62] Kim C.-S., Joe Y., Choi H.-S., Back S.H., Park J.W., Chung H.T., Roh E., Kim M.-S., Ha T.Y., Yu R. (2019). Deficiency of fibroblast growth factor 21 aggravates obesity-induced atrophic responses in skeletal muscle. J. Inflamm..

[63] Vaughan R.A., Gannon N.P., Barberena M.A., Garcia-Smith R., Bisoffi M., Mermier C.M., Conn C.A., Trujillo K.A. (2014). Characterization of the metabolic effects of irisin on skeletal muscle in vitro. Diabetes Obes. Metab..

[64] Boström P., Wu J., Jedrychowski M.P., Korde A., Ye L., Lo J.C., Rasbach K.A., Boström E.A., Choi J.H., Long J.Z. (2012). A PGC1-α-dependent myokine that drives brown-fat-like development of white fat and thermogenesis. Nature.

[65] Kurdiova T., Balaz M., Vician M., Maderova D., Vlcek M., Valkovic L., Srbecky M., Imrich R., Kyselovicova O., Belan V. (2014). Effects of obesity, diabetes and exercise on *Fndc5* gene expression and irisin release in human skeletal muscle and adipose tissue: In vivo and in vitro studies. J. Physiol..

[66] Wende A., Schaeffer P.J., Parker G.J., Zechner C., Han D.-H., Chen M.M., Hancock C., Lehman J.J., Huss J.M., McClain D. (2007). A Role for the Transcriptional Coactivator PGC-1α in Muscle Refueling. J. Biol. Chem..

[67] Scarpulla R.C. (2008). Transcriptional Paradigms in Mammalian Mitochondrial Biogenesis and Function. Physiol. Rev..

[68] Puigserver P., Spiegelman B.M. (2003). Peroxisome Proliferator-Activated Receptor-γ Coactivator 1α (PGC-1α): Transcriptional Coactivator and Metabolic Regulator. Endocr. Rev..

[69] Xiang L., Xiang G., Yue L., Zhang J., Zhao L. (2014). Circulating irisin levels are positively associated with endothelium-dependent vasodilation in newly diagnosed type 2 diabetic patients without clinical angiopathy. Atherosclerosis.

[70] Alis R., Sanchis-Gomar F., Pareja-Galeano H., Hernández-Mijares A., Romagnoli M., Víctor V.M., Rocha M. (2014). Association between irisin and homocysteine in euglycemic and diabetic subjects. Clin. Biochem..

[71] Yano N., Zhang L., Wei D., Dubielecka P.M., Wei L., Zhuang S., Zhu P., Qin G., Liu P.Y., Chin Y.E. (2020). Irisin counteracts high glucose and fatty acid-induced cytotoxicity by preserving the AMPK-insulin receptor signaling axis in C2C12 myoblasts. Am. J. Physiol. Metab..

[72] Zhang Y., Li R., Meng Y., Li S., Donelan W., Zhao Y., Qi L., Zhang M., Wang X., Cui T. (2014). Irisin Stimulates Browning of White Adipocytes Through Mitogen-Activated Protein Kinase p38 MAP Kinase and ERK MAP Kinase Signaling. Diabetes.

[73] Lee H.J., Lee J.O., Kim N., Kim J.K., Kim H.I., Lee Y.W., Kim S.J., Choi J.-I., Oh Y., Kim J.H. (2015). Irisin, a novel myokine, regulates glucose uptake in skeletal muscle cells via AMPK. Mol. Endocrinol..

[74] Kim H., Wrann C.D., Jedrychowski M., Vidoni S., Kitase Y., Nagano K., Zhou C., Chou J., Parkman V.A., Novick S.J. (2018). Irisin mediates effects on bone and fat via alphaV integrin receptors. Cell.

[75] Ghanemi A., Melouane A., Yoshioka M., St-Amand J. (2020). Exercise Training of Secreted Protein Acidic and Rich in Cysteine *(Sparc)* KO Mice Suggests That Exercise-Induced Muscle Phenotype Changes Are SPARC-Dependent. Appl. Sci..

[76] Aoi W., Hirano N., Lassiter D.G., Björnholm M., Chibalin A.V., Sakuma K., Tanimura Y., Mizushima K., Takagi T., Naito Y. (2019). Secreted protein acidic and rich in cysteine (SPARC) improves glucose tolerance *via* AMP-activated protein kinase activation. FASEB J..

[77] Bradshaw A.D., Graves D.C., Motamed K., Sage E.H. (2003). SPARC-null mice exhibit increased adiposity without significant differences in overall body weight. Proc. Natl. Acad. Sci. USA.

[78] Cho W.J., Kim E.J., Lee S.J., Kim H.D., Shin H.J., Lim W.K. (2000). Involvement of SPARC in in Vitro Differentiation of Skeletal Myoblasts. Biochem. Biophys. Res. Commun..

[79] Motamed K., Blake D.J., Angello J.C., Allen B.L., Rapraeger A.C., Hauschka S.D., Sage E.H. (2003). Fibroblast growth factor receptor-1 mediates the inhibition of endothelial cell proliferation and the promotion of skeletal myoblast differentiation by SPARC: A role for protein kinase A. J. Cell. Biochem..

[80] Nakamura S.K., Nakano S.-I., Miyoshi T., Yamanouchi K., Matsuwaki T., Nishihara M. (2012). Age-related resistance of skeletal muscle-derived progenitor cells to SPARC may explain a shift from myogenesis to adipogenesis. Aging.

[81] Shi C.-X., Zhao M.-X., Shu X.-D., Xiong X.-Q., Wang J.-J., Gao X.-Y., Chen Q., Li Y.-H., Kang Y.-M., Zhu G.-Q. (2016). β-aminoisobutyric acid attenuates hepatic endoplasmic reticulum stress and glucose/lipid metabolic disturbance in mice with type 2 diabetes. Sci. Rep..

[82] Jung T.W., Hwang H.-J., Hong H.C., Yoo H.J., Baik S.H., Choi K.M. (2015). BAIBA attenuates insulin resistance and inflammation induced by palmitate or a high fat diet via an AMPK–PPARδ-dependent pathway in mice. Diabetologia.

[83] Roberts L., Boström P., O’Sullivan J., Schinzel R.T., Lewis G.D., Dejam A., Lee Y.-K., Palma M.J., Calhoun S., Georgiadi A. (2014). β-Aminoisobutyric Acid Induces Browning of White Fat and Hepatic β-Oxidation and Is Inversely Correlated with Cardiometabolic Risk Factors. Cell Metab..

[84] Jung T.W., Park H.S., Choi G.H., Kim D., Lee T. (2018). β-aminoisobutyric acid attenuates LPS-induced inflammation and insulin resistance in adipocytes through AMPK-mediated pathway. J. Biomed. Sci..

[85] Tanianskii D.A., Jarzebska N., Birkenfeld A.L., O’Sullivan J.F., Rodionov R.N. (2019). Beta-Aminoisobutyric Acid as a Novel Regulator of Carbohydrate and Lipid Metabolism. Nutrients.

[86] Begriche K., Massart J., Abbey-Toby A., Igoudjil A., Lettéron P., Fromenty B. (2008). β-Aminoisobutyric Acid Prevents Diet-induced Obesity in Mice with Partial Leptin Deficiency. Obesity.

[87] Matthews V.B., Åström M.-B., Chan S., Bruce C., Krabbe K.S., Prelovsek O., Åkerström T., Yfanti C., Broholm C., Mortensen O.H. (2009). Brain-derived neurotrophic factor is produced by skeletal muscle cells in response to contraction and enhances fat oxidation via activation of AMP-activated protein kinase. Diabetologia.

[88] Matsumoto J., Takada S., Furihata T., Nambu H., Kakutani N., Maekawa S., Mizushima W., Nakano I., Fukushima A., Yokota T. (2021). Brain-Derived Neurotrophic Factor Improves Impaired Fatty Acid Oxidation Via the Activation of Adenosine Monophosphate-Activated Protein Kinase-α—Proliferator-Activated Receptor-r Coactivator-1α Signaling in Skeletal Muscle of Mice with Heart Failure. Circ. Heart Fail..

[89] Numakawa T., Suzuki S., Kumamaru E., Adachi N., Richards M., Kunugi H. (2010). BDNF function and intracellular signaling in neurons. Histol. Histopathol..

[90] Yang X., Brobst D., Chan W.S., Tse M.C.L., Herlea-Pana O., Ahuja P., Bi X., Zaw A.M., Kwong Z.S.W., Jia W.-H. (2019). Muscle-generated BDNF is a sexually dimorphic myokine that controls metabolic flexibility. Sci. Signal..

[91] Delezie J., Weihrauch M., Maier G., Tejero R., Ham D.J., Gill J.F., Karrer-Cardel B., Rüegg M.A., Tabares L., Handschin C. (2019). BDNF is a mediator of glycolytic fiber-type specification in mouse skeletal muscle. Proc. Natl. Acad. Sci. USA.

[92] Yamanaka M., Tsuchida A., Nakagawa T., Nonomura T., Ono-Kishino M., Sugaru E., Noguchi H., Taiji M. (2007). Brain-derived neurotrophic factor enhances glucose utilization in peripheral tissues of diabetic mice. Diabetes Obes. Metab..

[93] Kim H.-J., Higashimori T., Park S.-Y., Choi H., Dong J., Kim Y.-J., Noh H.-L., Cho Y.-R., Cline G., Kim Y.-B. (2004). Differential Effects of Interleukin-6 and -10 on Skeletal Muscle and Liver Insulin Action In Vivo. Diabetes.

[94] Ruderman N.B., Keller C., Richard A.-M., Saha A.K., Luo Z., Xiang X., Giralt M., Ritov V.B., Menshikova E.V., Kelley D.E. (2006). Interleukin-6 Regulation of AMP-Activated Protein Kinase: Potential Role in the Systemic Response to Exercise and Prevention of the Metabolic Syndrome. Diabetes.

[95] Wolsk E., Mygind H., Grøndahl T.S., Pedersen B.K., van Hall G. (2010). IL-6 selectively stimulates fat metabolism in human skeletal muscle. Am. J. Physiol. Metab..

[96] Senn J.J. (2006). Toll-like Receptor-2 Is Essential for the Development of Palmitate-induced Insulin Resistance in Myotubes. J. Biol. Chem..

[97] Jové M., Planavila A., Sánchez R.M., Merlos M., Laguna J.C., Vázquez-Carrera M. (2006). Palmitate Induces Tumor Necrosis Factor-α Expression in C2C12 Skeletal Muscle Cells by a Mechanism Involving Protein Kinase C and Nuclear Factor-κB Activation. Endocrinology.

[98] Foss-Freitas M.C., Foss N.T., Donadi E., Foss M.C. (2006). In Vitro TNF- and IL-6 Production by Adherent Peripheral Blood Mononuclear Cells Obtained from Type 1 and Type 2 Diabetic Patients Evaluated according to the Metabolic Control. Ann. N. Y. Acad. Sci..

[99] Carey A.L., Bruce C.R., Sacchetti M., Anderson M., Olsen D.B., Saltin B., Hawley J., Febbraio M.A. (2004). Interleukin-6 and tumor necrosis factor-? are not increased in patients with Type 2 diabetes: Evidence that plasma interleukin-6 is related to fat mass and not insulin responsiveness. Diabetologia.

[100] Broholm C., Pedersen B.K. (2010). Leukaemia inhibitory factor--an exercise-induced myokine. Exerc. Immunol. Rev..

[101] Broholm C., Laye M.J., Brandt C., Vadalasetty R., Pilegaard H., Pedersen B.K., Schéele C. (2011). LIF is a contraction-induced myokine stimulating human myocyte proliferation. J. Appl. Physiol..

[102] Brandt N., O’Neill H.M., Kleinert M., Schjerling P., Vernet E., Steinberg G.R., Richter E.A., Jorgensen S.B. (2015). Leukemia inhibitory factor increases glucose uptake in mouse skeletal muscle. Am. J. Physiol. Metab..

[103] Broholm C., Brandt C., Schultz N.S., Nielsen A.R., Pedersen B.K., Scheele C. (2012). Deficient leukemia inhibitory factor signaling in muscle precursor cells from patients with type 2 diabetes. Am. J. Physiol. Metab..

[104] Toledo-Corral C.M., Banner L.R. (2012). Early changes of LIFR and gp130 in sciatic nerve and muscle of diabetic mice. Acta Histochem..

[105] Grit E., Legård B.K.P. (2019). Muscle and Exercise Physiology.

[106] Tamura Y., Watanabe K., Kantani T., Hayashi J., Ishida N., Kaneki M. (2011). Upregulation of circulating IL-15 by treadmill running in healthy individuals: Is IL-15 an endocrine mediator of the beneficial effects of endurance exercise?. Endocr. J..

[107] Pierce J.R., Maples J., Hickner R.C. (2015). IL-15 concentrations in skeletal muscle and subcutaneous adipose tissue in lean and obese humans: Local effects of IL-15 on adipose tissue lipolysis. Am. J. Physiol. Metab..

[108] Bazgir B., Salesi M., Koushki M., Amirghofran Z. (2015). Effects of Eccentric and Concentric Emphasized Resistance Exercise on IL-15 Serum Levels and Its Relation to Inflammatory Markers in Athletes and Non-Athletes. Asian J. Sports Med..

[109] Barra N.G., Reid S., MacKenzie R., Werstuck G., Trigatti B.L., Richards C., Holloway A.C., Ashkar A.A. (2010). Interleukin-15 Contributes to the Regulation of Murine Adipose Tissue and Human Adipocytes. Obesity.

[110] Almendro V., Fuster G., Busquets S., Ametller E., Figueras M., Argiles J.M., López-Soriano F.J. (2008). Effects of IL-15 on Rat Brown Adipose Tissue: Uncoupling Proteins and PPARs. Obesity.

[111] Quinn L.S., Anderson B.G., Conner J.D., Pistilli E.E., Wolden-Hanson T. (2011). Overexpression of interleukin-15 in mice promotes resistance to diet-induced obesity, increased insulin sensitivity, and markers of oxidative skeletal muscle metabolism. Int. J. Interf. Cytokine Mediat. Res..

[112] Sun H., Liu D. (2015). Hydrodynamic delivery of interleukin 15 gene promotes resistance to high fat diet-induced obesity, fatty liver and improves glucose homeostasis. Gene Ther..

[113] Gray S.R., Kamolrat T. (2011). The effect of exercise induced cytokines on insulin stimulated glucose transport in C2C12 cells. Cytokine.

[114] Krolopp J.E., Thornton S.M., Abbott M.J. (2016). IL-15 Activates the Jak3/STAT3 Signaling Pathway to Mediate Glucose Uptake in Skeletal Muscle Cells. Front. Physiol..

[115] Quinn L.S., Anderson B.G., Conner J.D., Wolden-Hanson T. (2013). IL-15 Overexpression Promotes Endurance, Oxidative Energy Metabolism, and Muscle PPARδ, SIRT1, PGC-1α, and PGC-1β Expression in Male Mice. Endocrinology.

[116] Wong G.W., Wang J., Hug C., Tsao T.-S., Lodish H.F. (2004). A family of Acrp30/adiponectin structural and functional paralogs. Proc. Natl. Acad. Sci. USA.

[117] Peterson J.M., Aja S., Wei Z., Wong G.W. (2012). CTRP1 Protein Enhances Fatty Acid Oxidation via AMP-activated Protein Kinase (AMPK) Activation and Acetyl-CoA Carboxylase (ACC) Inhibition. J. Biol. Chem..

[118] Peterson J., Seldin M.M., Wei Z., Aja S., Wong G.W. (2013). CTRP3 attenuates diet-induced hepatic steatosis by regulating triglyceride metabolism. Am. J. Physiol. Liver Physiol..

[119] Lim S., Choi S.H., Koo B.K., Kang S.M., Yoon J.W., Jang H.C., Choi S.M., Lee M.G., Lee W., Shin H. (2012). Effects of Aerobic Exercise Training on C1q Tumor Necrosis Factor α-Related Protein Isoform 5 (Myonectin): Association with Insulin Resistance and Mitochondrial DNA Density in Women. J. Clin. Endocrinol. Metab..

[120] Seldin M.M., Lei X., Tan S.Y., Stanson K.P., Wei Z., Wong G.W. (2013). Skeletal Muscle-derived Myonectin Activates the Mammalian Target of Rapamycin (mTOR) Pathway to Suppress Autophagy in Liver. J. Biol. Chem..

[121] Raschke S., Eckel J. (2013). Adipo-Myokines: Two Sides of the Same Coin—Mediators of Inflammation and Mediators of Exercise. Mediat. Inflamm..

[122] Li K., Liao X., Wang K., Mi Q., Zhang T., Jia Y., Xu X., Luo X., Zhang C., Liu H. (2018). Myonectin Predicts the Development of Type 2 Diabetes. J. Clin. Endocrinol. Metab..

[123] Seldin M.M., Peterson J.M., Byerly M.S., Wei Z., Wong G.W. (2012). Myonectin (CTRP15), a Novel Myokine That Links Skeletal Muscle to Systemic Lipid Homeostasis. J. Biol. Chem..

[124] Pourranjbar M., Arabnejad N., Naderipour K., Rafie F. (2018). Effects of Aerobic Exercises on Serum Levels of Myonectin and Insulin Resistance in Obese and Overweight Women. J. Med. Life.

[125] Lenk K., Schur R., Linke A., Erbs S., Matsumoto Y., Adams V., Schuler G. (2009). Impact of exercise training on myostatin expression in the myocardium and skeletal muscle in a chronic heart failure model. Eur. J. Heart Fail..

[126] Joulia D., Bernardi H., Garandel V., Rabenoelina F., Vernus B., Cabello G. (2003). Mechanisms involved in the inhibition of myoblast proliferation and differentiation by myostatin. Exp. Cell Res..

[127] Amthor H., Macharia R., Navarrete R., Schuelke M., Brown S.C., Otto A., Voit T., Muntoni F., Vrbóva G., Partridge T. (2007). Lack of myostatin results in excessive muscle growth but impaired force generation. Proc. Natl. Acad. Sci. USA.

[128] McPherron A.C., Lee S.-J. (1997). Double muscling in cattle due to mutations in the myostatin gene. Proc. Natl. Acad. Sci. USA.

[129] McPherron A., Lawler A.M., Lee S.-J. (1997). Regulation of skeletal muscle mass in mice by a new TGF-p superfamily member. Nature.

[130] Sartori R., Milan G., Patron M., Mammucari C., Blaauw B., Abraham R., Sandri M. (2009). Smad2 and 3 transcription factors control muscle mass in adulthood. Am. J. Physiol. Physiol..

[131] Sriram S., Subramanian S., Sathiakumar D., Venkatesh R., Salerno M.S., McFarlane C.D., Kambadur R., Sharma M. (2011). Modulation of reactive oxygen species in skeletal muscle by myostatin is mediated through NF-κB. Aging Cell.

[132] McPherron A.C., Lee S.-J. (2002). Suppression of body fat accumulation in myostatin-deficient mice. J. Clin. Investig..

[133] Lehr S., Hartwig S., Sell H. (2012). Adipokines: A treasure trove for the discovery of biomarkers for metabolic disorders. Proteom. Clin. Appl..

[134] Lin J., Arnold H.B., Della-Fera M.A., Azain M., Hartzell D.L., Baile C.A. (2002). Myostatin Knockout in Mice Increases Myogenesis and Decreases Adipogenesis. Biochem. Biophys. Res. Commun..

[135] Wilkes J.J., Lloyd D.J., Gekakis N. (2009). Loss-of-Function Mutation in Myostatin Reduces Tumor Necrosis Factor α Production and Protects Liver Against Obesity-Induced Insulin Resistance. Diabetes.

[136] Zhao B., Wall R.J., Yang J. (2005). Transgenic expression of myostatin propeptide prevents diet-induced obesity and insulin resistance. Biochem. Biophys. Res. Commun..

[137] Guo T., Jou W., Chanturiya T., Portas J., Gavrilova O., McPherron A.C. (2009). Myostatin Inhibition in Muscle, but Not Adipose Tissue, Decreases Fat Mass and Improves Insulin Sensitivity. PLoS ONE.

[138] Hamrick M.W., Pennington C., Webb C.N., Isales C.M. (2006). Resistance to body fat gain in ‘double-muscled’ mice fed a high-fat diet. Int. J. Obes..

[139] Cleasby M.E., Jarmin S., Eilers W., Elashry M., Andersen D.K., Dickson G., Foster K. (2014). Local overexpression of the myostatin propeptide increases glucose transporter expression and enhances skeletal muscle glucose disposal. Am. J. Physiol. Metab..

[140] Ellingsgaard H., Hauselmann I., Schuler B., Habib A.M., Baggio L.L., Zeman-Meier D., Eppler E., Bouzakri K., Wueest S., Muller Y. (2011). Interleukin-6 enhances insulin secretion by increasing glucagon-like peptide-1 secretion from L cells and alpha cells. Nat. Med..

[141] Handschin C., Choi C.S., Chin S., Kim S., Kawamori D., Kurpad A.J., Neubauer N., Hu J., Mootha V.K., Kim Y.-B. (2007). Abnormal glucose homeostasis in skeletal muscle–specific PGC-1α knockout mice reveals skeletal muscle–pancreatic β cell crosstalk. J. Clin. Investig..

[142] Hirner S., Krohne C., Schuster A., Hoffmann S., Witt S., Erber R., Sticht C., Gasch A., Labeit S., Labeit D. (2008). MuRF1-dependent Regulation of Systemic Carbohydrate Metabolism as Revealed from Transgenic Mouse Studies. J. Mol. Biol..

[143] Pedersen B.K., Febbraio M.A. (2008). Muscle as an Endocrine Organ: Focus on Muscle-Derived Interleukin-6. Physiol. Rev..

[144] Scheler M., Irmler M., Lehr S., Hartwig S., Staiger H., Al-Hasani H., Beckers J., de Angelis M.H., Häring H.-U., Weigert C. (2013). Cytokine response of primary human myotubes in an in vitro exercise model. Am. J. Physiol. Physiol..

[145] Wang Z., Oh E., Clapp D.W., Chernoff J., Thurmond D.C. (2011). Inhibition or Ablation of p21-activated Kinase (PAK1) Disrupts Glucose Homeostatic Mechanisms in Vivo. J. Biol. Chem..

[146] Tunduguru R., Zhang J., Aslamy A., Salunkhe V.A., Brozinick J.T., Elmendorf J.S., Thurmond D.C. (2017). The actin-related p41ARC subunit contributes to p21-activated kinase-1 (PAK1)–mediated glucose uptake into skeletal muscle cells. J. Biol. Chem..

[147] Zhang W., Wu Y., Du L., Tang D.D., Gunst S.J. (2005). Activation of the Arp2/3 complex by N-WASp is required for actin polymerization and contraction in smooth muscle. Am. J. Physiol. Physiol..

[148] Merz K.E., Tunduguru R., Ahn M., Salunkhe V.A., Veluthakal R., Hwang J., Bhattacharya S., McCown E.M., Garcia P.A., Zhou C. (2022). Changes in Skeletal Muscle PAK1 Levels Regulate Tissue Crosstalk to Impact Whole Body Glucose Homeostasis. Front. Endocrinol..

[149] Ryan A.J., Ciaraldi T.P., Henry R.R. (2019). Myokine Regulation of Insulin Secretion: Impact of Inflammation and Type 2 Diabetes. Front. Physiol..

[150] Schulthess F.T., Paroni F., Sauter N.S., Shu L., Ribaux P., Haataja L., Strieter R.M., Oberholzer J., King C.C., Maedler K. (2009). CXCL10 Impairs β Cell Function and Viability in Diabetes through TLR4 Signaling. Cell Metab..

[151] Lee E.Y., Lee Z.-H., Song Y.W. (2009). CXCL10 and autoimmune diseases. Autoimmun. Rev..

[152] Nigi L., Brusco N., Grieco G.E., Licata G., Krogvold L., Marselli L., Gysemans C., Overbergh L., Marchetti P., Mathieu C. (2020). Pancreatic Alpha-Cells Contribute Together with Beta-Cells to CXCL10 Expression in Type 1 Diabetes. Front. Endocrinol..

[153] Nicoletti F., Conget I., Di Mauro M., Di Marco R., Mazzarino M.C., Bendtzen K., Messina A., Gomis R. (2002). Serum concentrations of the interferon-γ-inducible chemokine IP-10/CXCL10 are augmented in both newly diagnosed Type I diabetes mellitus patients and subjects at risk of developing the disease. Diabetologia.

[154] Rhode A., Pauza M.E., Barral A.M., Rodrigo E., Oldstone M.B.A., Von Herrath M.G., Christen U. (2005). Islet-Specific Expression of CXCL10 Causes Spontaneous Islet Infiltration and Accelerates Diabetes Development. J. Immunol..

[155] Lee S.-J. (2007). Quadrupling Muscle Mass in Mice by Targeting TGF-ß Signaling Pathways. PLoS ONE.

[156] Medeiros E.F., Phelps M.P., Fuentes F.D., Bradley T.M. (2009). Overexpression of follistatin in trout stimulates increased muscling. Am. J. Physiol. Integr. Comp. Physiol..

[157] Hansen J., Rinnov A., Krogh-Madsen R., Fischer C.P., Andreasen A.S., Berg R.M.G., Møller K., Pedersen B.K., Plomgaard P. (2013). Plasma follistatin is elevated in patients with type 2 diabetes: Relationship to hyperglycemia, hyperinsulinemia, and systemic low-grade inflammation. Diabetes/Metabolism Res. Rev..

[158] Yndestad A., Haukeland J.W., Dahl T.B., Bjøro K., Gladhaug I.P., Berge C., Damås J.K., Haaland T., Løberg E.M., Linnestad P. (2009). A Complex Role of Activin A in Non-Alcoholic Fatty Liver Disease. Am. J. Gastroenterol..

[159] Plomgaard P., Halban P.A., Bouzakri K. (2012). Bimodal impact of skeletal muscle on pancreatic β-cell function in health and disease. Diabetes Obes. Metab..

[160] Hansen J.S., Rutti S., Arous C., Clemmesen J.O., Secher N.H., Drescher A., Gonelle-Gispert C., Halban P.A., Pedersen B.K., Weigert C. (2016). Circulating Follistatin Is Liver-Derived and Regulated by the Glucagon-to-Insulin Ratio. J. Clin. Endocrinol. Metab..

[161] Bertolino P., Holmberg R., Reissmann E., Andersson O., Berggren P.-O., Ibáñez C.F. (2008). Activin B receptor ALK7 is a negative regulator of pancreatic β-cell function. Proc. Natl. Acad. Sci. USA.

[162] Ripoche D., Charbord J., Hennino A., Teinturier R., Bonnavion R., Jaafar R., Goehrig D., Cordier-Bussat M., Ritvos O., Zhang C.X. (2015). ActivinB Is Induced in Insulinoma to Promote Tumor Plasticity through a β-Cell-Induced Dedifferentiation. Mol. Cell. Biol..

[163] Jedrychowski M.P., Wrann C.D., Paulo J.A., Gerber K.K., Szpyt J., Robinson M.M., Sreekumaran Nair K., Gygi S.P., Spiegelman B.M. (2015). Detection and Quantitation of Circulating Human Irisin by Tandem Mass Spectrometry. Cell Metab..

[164] Liu S., Du F., Li X., Wang M., Duan R., Zhang J., Wu Y., Zhang Q. (2017). Effects and underlying mechanisms of irisin on the proliferation and apoptosis of pancreatic β cells. PLoS ONE.

[165] Natalicchio A., Marrano N., Biondi G., Spagnuolo R., Labarbuta R., Porreca I., Cignarelli A., Bugliani M., Marchetti P., Perrini S. (2017). The Myokine Irisin Is Released in Response to Saturated Fatty Acids and Promotes Pancreatic β-Cell Survival and Insulin Secretion. Diabetes.

[166] Catoire M., Mensink M., Kalkhoven E., Schrauwen P., Kersten S. (2014). Identification of human exercise-induced myokines using secretome analysis. Physiol. Genom..

[167] Lee Y.S., Morinaga H., Kim J.J., Lagakos W., Taylor S., Keshwani M., Perkins G., Dong H., Kayali A.G., Sweet I.R. (2013). The Fractalkine/CX3CR1 System Regulates β Cell Function and Insulin Secretion. Cell.

[168] Riopel M., Seo J.B., Bandyopadhyay G.K., Li P., Wollam J., Chung H., Jung S.-R., Murphy A., Wilson M., De Jong R. (2018). Chronic fractalkine administration improves glucose tolerance and pancreatic endocrine function. J. Clin. Investig..

[169] Rutti S., Arous C., Schvartz D., Timper K., Sanchez J.-C., Dermitzakis E., Donath M.Y., Halban P.A., Bouzakri K. (2014). Fractalkine (CX3CL1), a new factor protecting β-cells against TNFα. Mol. Metab..

[170] Mao X., Kikani C.K., Riojas R.A., Langlais P., Wang L., Ramos F.J., Fang Q., Christ-Roberts C.Y., Hong J.Y., Kim R.Y. (2006). APPL1 binds to adiponectin receptors and mediates adiponectin signalling and function. Nat. Cell Biol..

[171] Yamauchi T., Kadowaki T. (2013). Adiponectin Receptor as a Key Player in Healthy Longevity and Obesity-Related Diseases. Cell Metab..

[172] Schinzari F., Veneziani A., Mores N., Barini A., Di Daniele N., Cardillo C., Tesauro M. (2017). Beneficial Effects of Apelin on Vascular Function in Patients with Central Obesity. Hypertension.

[173] He S., Li J., Wang J., Zhang Y. (2019). Hypoxia exposure alleviates impaired muscular metabolism, glucose tolerance, and aerobic capacity in apelin-knockout mice. FEBS Open Bio.

[174] Zhu S., Sun F., Li W., Cao Y., Wang C., Wang Y., Liang D., Zhang R., Zhang S., Wang H. (2011). Apelin stimulates glucose uptake through the PI3K/Akt pathway and improves insulin resistance in 3T3-L1 adipocytes. Mol. Cell. Biochem..

[175] Ceylan-Isik A.F., Kandadi M.R., Xu X., Hua Y., Chicco A.J., Ren J., Nair S. (2013). Apelin administration ameliorates high fat diet-induced cardiac hypertrophy and contractile dysfunction. J. Mol. Cell. Cardiol..

[176] Pedersen L., Olsen C.H., Pedersen B.K., Hojman P. (2012). Muscle-derived expression of the chemokine CXCL1 attenuates diet-induced obesity and improves fatty acid oxidation in the muscle. Am. J. Physiol. Metab..

[177] Li H., Wu G., Fang Q., Zhang M., Hui X., Sheng B., Wu L., Bao Y., Li P., Xu A. (2018). Fibroblast growth factor 21 increases insulin sensitivity through specific expansion of subcutaneous fat. Nat. Commun..

[178] Inagaki T., Dutchak P., Zhao G., Ding X., Gautron L., Parameswara V., Li Y., Goetz R., Mohammadi M., Esser V. (2007). Endocrine Regulation of the Fasting Response by PPARα-Mediated Induction of Fibroblast Growth Factor 21. Cell Metab..

[179] Zhao C., Liu Y., Xiao J., Liu L., Chen S., Mohammadi M., McClain C.J., Li X., Feng W. (2015). FGF21 mediates alcohol-induced adipose tissue lipolysis by activation of systemic release of catecholamine in mice. J. Lipid Res..

[180] Chau M.D.L., Gao J., Yang Q., Wu Z., Gromada J. (2010). Fibroblast growth factor 21 regulates energy metabolism by activating the AMPK–SIRT1–PGC-1α pathway. Proc. Natl. Acad. Sci. USA.

[181] Schlein C., Talukdar S., Heine M., Fischer A.W., Krott L.M., Nilsson S.K., Brenner M.B., Heeren J., Scheja L. (2016). FGF21 Lowers Plasma Triglycerides by Accelerating Lipoprotein Catabolism in White and Brown Adipose Tissues. Cell Metab..

[182] Gimeno R.E., Moller D.E. (2014). FGF21-based pharmacotherapy—Potential utility for metabolic disorders. Trends Endocrinol. Metab..

[183] Nadeau L., Patten D., Caron A., Garneau L., Pinault-Masson E., Foretz M., Haddad P., Anderson B., Quinn L., Jardine K. (2018). IL-15 improves skeletal muscle oxidative metabolism and glucose uptake in association with increased respiratory chain supercomplex formation and AMPK pathway activation. Biochim. Biophys. Acta (BBA)-Gen. Subj..

[184] Timper K., Denson J.L., Steculorum S., Heilinger C., Ruud L.E., Wunderlich C.M., Rose-John S., Wunderlich F.T., Brüning J.C. (2017). IL-6 Improves Energy and Glucose Homeostasis in Obesity via Enhanced Central IL-6 trans -Signaling. Cell Rep..

[185] Jiang L.Q., Duque-Guimaraes D.E., Machado U.F., Zierath J.R., Krook A. (2013). Altered Response of Skeletal Muscle to IL-6 in Type 2 Diabetic Patients. Diabetes.

[186] Al-Khalili L., Bouzakri K., Glund S., Lönnqvist F., Koistinen H., Krook A. (2006). Signaling Specificity of Interleukin-6 Action on Glucose and Lipid Metabolism in Skeletal Muscle. Mol. Endocrinol..

[187] Hong E.-G., Ko H.J., Cho Y.-R., Kim H.-J., Ma Z., Yu T.Y., Friedline R.H., Kurt-Jones E., Finberg R., Fischer M.A. (2009). Interleukin-10 Prevents Diet-Induced Insulin Resistance by Attenuating Macrophage and Cytokine Response in Skeletal Muscle. Diabetes.

[188] Dagdeviren S., Jung D.Y., Friedline R.H., Noh H.L., Kim J.H., Patel P.R., Tsitsilianos N., Inashima K., Tran D.A., Hu X. (2017). IL-10 prevents aging-associated inflammation and insulin resistance in skeletal muscle. FASEB J..

[189] Xin C., Liu J., Zhang J.D., Zhu D., Wang H., Xiong L., Lee Y., Ye J., Lian K., Xu C. (2016). Irisin improves fatty acid oxidation and glucose utilization in type 2 diabetes by regulating the AMPK signaling pathway. Int. J. Obes..

[190] Lee J.O., Byun W.S., Kang M.J., Han J.A., Moon J., Shin M., Lee H.J., Chung J.H., Lee J., Son C. (2020). The myokine meteorin-like (metrnl) improves glucose tolerance in both skeletal muscle cells and mice by targeting AMPKα2. FEBS J..

[191] Hu W., Wang R., Sun B. (2021). Meteorin-Like Ameliorates β Cell Function by Inhibiting β Cell Apoptosis of and Promoting β Cell Proliferation via Activating the WNT/β-Catenin Pathway. Front. Pharmacol..

[192] Wei Z., Peterson J.M., Lei X., Cebotaru L., Wolfgang M.J., Baldeviano G.C., Wong G.W. (2012). C1q/TNF-related Protein-12 (CTRP12), a Novel Adipokine That Improves Insulin Sensitivity and Glycemic Control in Mouse Models of Obesity and Diabetes. J. Biol. Chem..

[193] Liu X.-H., Bauman W.A., Cardozo C.P. (2018). Myostatin inhibits glucose uptake via suppression of insulin-dependent and -independent signaling pathways in myoblasts. Physiol. Rep..

[194] Hittel D.S., Axelson M., Sarna N., Shearer J., Huffman K.M., Kraus W.E. (2010). Myostatin Decreases with Aerobic Exercise and Associates with Insulin Resistance. Med. Sci. Sports Exerc..

[195] Allen D.L., Cleary A.S., Speaker K.J., Lindsay S.F., Uyenishi J., Reed J.M., Madden M.C., Mehan R.S. (2008). Myostatin, activin receptor IIb, and follistatin-like-3 gene expression are altered in adipose tissue and skeletal muscle of obese mice. Am. J. Physiol. Metab..

[196] Lee N., Ali N., Zhang L., Qi Y., Clarke I., Enriquez R., Brzozowska M., Lee I., Rogers M., Laybutt D. (2018). Osteoglycin, a novel coordinator of bone and glucose homeostasis. Mol. Metab..

[197] Kim S.P., Li Z., Zoch M.L., Frey J.L., Bowman C.E., Kushwaha P., Ryan K.A., Goh B., Scafidi S., Pickett J.E. (2017). Fatty acid oxidation by the osteoblast is required for normal bone acquisition in a sex- and diet-dependent manner. JCI Insight.

[198] Nie J., Sage E.H. (2009). SPARC Inhibits Adipogenesis by Its Enhancement of β-Catenin Signaling. J. Biol. Chem..

[199] Knudsen J.G., Murholm M., Carey A.L., Biensø R.S., Basse A.L., Allen T.L., Hidalgo J., Kingwell B.A., Febbraio M.A., Hansen J.B. (2014). Role of IL-6 in Exercise Training- and Cold-Induced UCP1 Expression in Subcutaneous White Adipose Tissue. PLoS ONE.

[200] Wan Z., Ritchie I., Beaudoin M.-S., Castellani L., Chan C.B., Wright D.C. (2012). IL-6 Indirectly Modulates the Induction of Glyceroneogenic Enzymes in Adipose Tissue during Exercise. PLoS ONE.

[201] Van Hall G., Steensberg A., Sacchetti M., Fischer C., Keller C., Schjerling P., Hiscock N., Moller K., Saltin B., Febbraio M.A. (2003). Interleukin-6 Stimulates Lipolysis and Fat Oxidation in Humans. J. Clin. Endocrinol. Metab..

[202] Wueest S., Konrad D. (2018). The role of adipocyte-specific IL-6-type cytokine signaling in FFA and leptin release. Adipocyte.

[203] Javaid H.M.A., Sahar N.E., ZhuGe D.-L., Huh J.Y. (2021). Exercise Inhibits NLRP3 Inflammasome Activation in Obese Mice via the Anti-Inflammatory Effect of Meteorin-like. Cells.

[204] Rao R.R., Long J.Z., White J.P., Svensson K.J., Lou J., Lokurkar I., Jedrychowski M.P., Ruas J.L., Wrann C.D., Lo J.C. (2014). Meteorin-like Is a Hormone that Regulates Immune-Adipose Interactions to Increase Beige Fat Thermogenesis. Cell.

[205] Singh R., Pervin S., Lee S.-J., Kuo A., Grijalva V., David J., Vergnes L., Reddy S.T. (2018). Metabolic profiling of follistatin overexpression: A novel therapeutic strategy for metabolic diseases. Diabetes Metab. Syndr. Obes. Targets Ther..

[206] Braga M., Reddy S.T., Vergnes L., Pervin S., Grijalva V., Stout D., David J., Li X., Tomasian V., Reid C.B. (2014). Follistatin promotes adipocyte differentiation, browning, and energy metabolism. J. Lipid Res..

[207] Hou N., Liu Y., Han F., Wang D., Hou X., Hou S., Sun X. (2016). Irisin improves perivascular adipose tissue dysfunction via regulation of the heme oxygenase-1/adiponectin axis in diet-induced obese mice. J. Mol. Cell. Cardiol..

[208] Irving B.A., Still C.D., Argyropoulos G. (2014). Does IRISIN Have a BRITE Future as a Therapeutic Agent in Humans?. Curr. Obes. Rep..

[209] Manni L., Nikolova V., Vyagova D., Chaldakov G.N., Aloe L. (2005). Reduced plasma levels of NGF and BDNF in patients with acute coronary syndromes. Int. J. Cardiol..

[210] Krabbe K.S., Nielsen A.R., Krogh-Madsen R., Plomgaard P., Rasmussen P., Erikstrup C., Fischer C., Lindegaard B., Petersen A.M.W., Taudorf S. (2007). Brain-derived neurotrophic factor (BDNF) and type 2 diabetes. Diabetologia.

[211] Huang E.J., Reichardt L.F. (2001). Neurotrophins: Roles in Neuronal Development and Function. Annu. Rev. Neurosci..

[212] Ono M., Ichihara J., Nonomura T., Itakura Y., Taiji M., Nakayama C., Noguchi H. (1997). Brain-Derived Neurotrophic Factor Reduces Blood Glucose Level in Obese Diabetic Mice but Not in Normal Mice. Biochem. Biophys. Res. Commun..

[213] Rios M., Fan G., Fekete C., Kelly J., Bates B., Kuehn R., Lechan R.M., Jaenisch R. (2001). Conditional Deletion of Brain-Derived Neurotrophic Factor in the Postnatal Brain Leads to Obesity and Hyperactivity. Mol. Endocrinol..

[214] Oelmann S., Nauck M., Völzke H., Bahls M., Friedrich N. (2016). Circulating Irisin Concentrations Are Associated with a Favourable Lipid Profile in the General Population. PLoS ONE.

[215] Moon H.Y., Becke A., Berron D., Becker B., Sah N., Benoni G., Janke E., Lubejko S., Greig N.H., Mattison J.A. (2016). Running-Induced Systemic Cathepsin B Secretion Is Associated with Memory Function. Cell Metab..

[216] Febbraio M.A., Hiscock N., Sacchetti M., Fischer C.P., Pedersen B.K. (2004). Interleukin-6 Is a Novel Factor Mediating Glucose Homeostasis During Skeletal Muscle Contraction. Diabetes.

[217] Peppler W.T., Townsend L.K., Meers G.M., Panasevich M.R., MacPherson R.E.K., Rector R.S., Wright D.C. (2019). Acute administration of IL-6 improves indices of hepatic glucose and insulin homeostasis in lean and obese mice. Am. J. Physiol. Liver Physiol..

[218] Tang H., Pang S., Wang M., Xiao X., Rong Y., Wang H., Zang Y.Q. (2010). TLR4 Activation Is Required for IL-17–Induced Multiple Tissue Inflammation and Wasting in Mice. J. Immunol..

[219] Duzova H., Karakoc Y., Emre M.H., Dogan Z.Y., Kilinc E. (2009). Effects of Acute Moderate and Strenuous Exercise Bouts on IL-17 Production and Inflammatory Response in Trained Rats. J. Sports Sci. Med..

[220] Harley I.T., Stankiewicz T.E., Giles D.A., Softic S., Flick L.M., Cappelletti M., Sheridan R., Xanthakos S.A., Steinbrecher K.A., Sartor R.B. (2014). IL-17 signaling accelerates the progression of nonalcoholic fatty liver disease in mice. Hepatology.

[221] Tarantino G., Costantini S., Finelli C., Capone F., Guerriero E., La Sala N., Gioia S., Castello G. (2014). Is serum Interleukin-17 associated with early atherosclerosis in obese patients?. J. Transl. Med..

[222] Cheng X., Yu X., Ding Y.-J., Fu Q.-Q., Xie J.-J., Tang T.-T., Yao R., Chen Y., Liao Y.-H. (2008). The Th17/Treg imbalance in patients with acute coronary syndrome. Clin. Immunol..

[223] Zhao M., Zhou X., Yuan C., Li R., Ma Y., Tang X. (2020). Association between serum irisin concentrations and sarcopenia in patients with liver cirrhosis: A cross-sectional study. Sci. Rep..

[224] Hu J., Ke Y., Wu F., Liu S., Ji C., Zhu X., Zhang Y. (2020). Circulating Irisin Levels in Patients with Nonalcoholic Fatty Liver Disease: A Systematic Review and Meta-Analysis. Gastroenterol. Res. Pr..

[225] Liu T.-Y., Shi C.-X., Gao R., Sun H.-J., Xiong X.-Q., Ding L., Chen Q., Li Y.-H., Wang J.-J., Kang Y.-M. (2015). Irisin inhibits hepatic gluconeogenesis and increases glycogen synthesis via the PI3K/Akt pathway in type 2 diabetic mice and hepatocytes. Clin. Sci..

[226] Liu M., Cao H., Hou Y., Sun G., Li D., Wang W. (2018). Liver Plays a Major Role in FGF-21 Mediated Glucose Homeostasis. Cell. Physiol. Biochem..

[227] Kim J.-S., Lee Y.-H., Yi H.-K. (2016). Gradual downhill running improves age-related skeletal muscle and bone weakness: Implication of autophagy and bone morphogenetic proteins. Exp. Physiol..

[228] Garneau L., Aguer C. (2019). Role of myokines in the development of skeletal muscle insulin resistance and related metabolic defects in type 2 diabetes. Diabetes Metab..

[229] Ouchi N., Ohashi K., Shibata R., Murohara T. (2016). Protective Roles of Adipocytokines and Myokines in Cardiovascular Disease. Circ. J..

[230] Ebert T., Kralisch S. (2016). Newly discovered myokines in chronic kidney disease. Pol. Arch. Intern. Med..

[231] Barbalho S.M., Flato U.A.P., Tofano R.J., Goulart R.D.A., Guiguer E.L., Detregiachi C.R.P., Buchaim D.V., Araújo A.C., Buchaim R.L., Reina F.T.R. (2020). Physical Exercise and Myokines: Relationships with Sarcopenia and Cardiovascular Complications. Int. J. Mol. Sci..

[232] Otaka N., Shibata R., Ohashi K., Uemura Y., Kambara T., Enomoto T., Ogawa H., Ito M., Kawanishi H., Maruyama S. (2018). Myonectin Is an Exercise-Induced Myokine That Protects the Heart from Ischemia-Reperfusion Injury. Circ. Res..

[233] Peng H., Wang Q., Lou T., Qin J., Jung S., Shetty V., Li F., Wang Y., Feng X.-H., Mitch W.E. (2017). Myokine mediated muscle-kidney crosstalk suppresses metabolic reprogramming and fibrosis in damaged kidneys. Nat. Commun..

[234] Colaianni G., Cuscito C., Mongelli T., Pignataro P., Buccoliero C., Liu P., Lu P., Sartini L., Di Comite M., Mori G. (2015). The myokine irisin increases cortical bone mass. Proc. Natl. Acad. Sci. USA.

[235] Kirk B., Feehan J., Lombardi G., Duque G. (2020). Muscle, Bone, and Fat Crosstalk: The Biological Role of Myokines, Osteokines, and Adipokines. Curr. Osteoporos. Rep..

[236] Kaji H. (2016). Effects of myokines on bone. BoneKEy Rep..

[237] Narendran P., Jackson N., Daley A., Thompson D., Stokes K., Greenfield S., Charlton M., Curran M., Solomon T., Nouwen A. (2017). Exercise to preserve β-cell function in recent-onset Type 1 diabetes mellitus (EXTOD)—A randomized controlled pilot trial. Diabet. Med..

[238] Paula F.M.M., Leite N.C., Vanzela E.C., Kurauti M.A., Freitas-Dias R., Carneiro E.M., Boschero A.C., Zoppi C.C. (2015). Exercise increases pancreatic β-cell viability in a model of type 1 diabetes through IL-6 signaling. FASEB J..

[239] Camporez J.P.G., Jornayvaz F., Petersen M.C., Pesta D., Guigni B., Serr J., Zhang D., Kahn M., Samuel V.T., Jurczak M. (2013). Cellular Mechanisms by Which FGF21 Improves Insulin Sensitivity in Male Mice. Endocrinology.

[240] Cuevas-Ramos D., Aguilar-Salinas C.A., Gómez-Pérez F.J. (2012). Metabolic actions of fibroblast growth factor 21. Curr. Opin. Pediatr..

[241] Li Z., Yang Y.-L., Zhu Y.-J., Li C.-G., Tang Y.-Z., Ni C.-L., Chen L.-M., Niu W.-Y. (2021). Circulating Serum Myonectin Levels in Obesity and Type 2 Diabetes Mellitus. Exp. Clin. Endocrinol. Diabetes.

[242] Zhang L., Rajan V., Lin E., Hu Z., Han H.Q., Zhou X., Song Y., Min H., Wang X., Du J. (2011). Pharmacological inhibition of myostatin suppresses systemic inflammation and muscle atrophy in mice with chronic kidney disease. FASEB J..

[243] Barlow J.P., Solomon T.P. (2018). Do skeletal muscle-secreted factors influence the function of pancreatic β-cells?. Am. J. Physiol. Metab..

[244] Ying L., Zhang Q., Yang Y.-M., Zhou J.-Y. (2022). A Combination of Serum Biomarkers in Elderly Patients with Sarcopenia: A Cross-Sectional Observational Study. Int. J. Endocrinol..

[245] Raschke S., Elsen M., Gassenhuber H., Sommerfeld M., Schwahn U., Brockmann B., Jung R., Wisloff U., Tjonna A.E., Raastad T. (2013). Evidence against a beneficial effect of irisin in humans. PLoS ONE.

[246] Erickson H.P. (2013). Irisin and FNDC5 in retrospect. Adipocyte.

[247] Geng L., Lam K.S.L., Xu A. (2020). The therapeutic potential of FGF21 in metabolic diseases: From bench to clinic. Nat. Rev. Endocrinol..

[248] Kaufman A., Abuqayyas L., Denney W., Tillman E., Rolph T. (2020). AKR-001, an Fc-FGF21 Analog, Showed Sustained Pharmacodynamic Effects on Insulin Sensitivity and Lipid Metabolism in Type 2 Diabetes Patients. Cell Rep. Med..

